# Anti-Cancer Mechanisms of Diarylpentanoid MS17 (1,5-Bis(2-hydroxyphenyl)-1,4-pentadiene-3-one) in Human Colon Cancer Cells: A Proteomics Approach

**DOI:** 10.3390/ijms25063503

**Published:** 2024-03-20

**Authors:** Kha Wai Hon, Syafiq Asnawi Zainal Abidin, Faridah Abas, Iekhsan Othman, Rakesh Naidu

**Affiliations:** 1 Jeffrey Cheah School of Medicine and Health Sciences, Monash University Malaysia, Jalan Lagoon Selatan, Bandar Sunway 47500, Selangor Darul Ehsan, Malaysia; hon.khawai@monash.edu (K.W.H.); syafiq.asnawi@monash.edu (S.A.Z.A.); iekhsan.othman@monash.edu (I.O.); 2 Natural Medicine and Product Research Laboratory, Institute of Bioscience, University Putra Malaysia, Serdang 43400, Selangor Darul Ehsan, Malaysia; faridah_abas@upm.edu.my; 3 Department of Food Science, Faculty of Food Science and Technology, University Putra Malaysia, Serdang 43400, Selangor Darul Ehsan, Malaysia

**Keywords:** colon cancer, diarylpentanoid, apoptosis, proteomics, molecular pathways, anti-proliferation

## Abstract

Diarylpentanoids are synthesized to overcome curcumin’s poor bioavailability and low stability to show enhanced anti-cancer effects. Little is known about the anti-cancer effects of diarylpentanoid MS17 (1,5-bis(2-hydroxyphenyl)-1,4-pentadiene-3-one) in colon cancer cells. This study aimed to elucidate molecular mechanisms and pathways modulated by MS17 in colon cancer based on proteomic profiling of primary SW480 and metastatic SW620 colon cancer cells. Cytotoxicity and apoptotic effects of MS17 were investigated using MTT assay, morphological studies, and Simple Western analysis. Proteomic profiling using LC/MS analysis identified differentially expressed proteins (DEPs) in MS17-treated cells, with further analysis in protein classification, gene ontology enrichment, protein–protein interaction network and Reactome pathway analysis. MS17 had lower EC_50_ values (SW480: 4.10 µM; SW620: 2.50 µM) than curcumin (SW480: 17.50 µM; SW620: 13.10 µM) with a greater anti-proliferative effect. MS17 treatment of 1× EC_50_ induced apoptotic changes in the morphology of SW480 and SW620 cells upon 24 h treatment. A total of 24 and 92 DEPs (fold change ≥ 1.50) were identified in SW480 and SW620 cells, respectively, upon MS17 treatment of 2× EC_50_ for 24 h. Pathway analysis showed that MS17 may induce its anti-cancer effects in both cells via selected DEPs associated with the top enriched molecular pathways. RPL and RPS ribosomal proteins, heat shock proteins (HSPs) and ubiquitin–protein ligases (UBB and UBC) were significantly associated with cellular responses to stress in SW480 and SW620 cells. Our findings suggest that MS17 may facilitate the anti-proliferative and apoptotic activities in primary (SW480) and metastatic (SW620) human colon cancer cells via the cellular responses to stress pathway. Further investigation is essential to determine the alternative apoptotic mechanisms of MS17 that are independent of caspase-3 activity and Bcl-2 protein expression in these cells. MS17 could be a potential anti-cancer agent in primary and metastatic colon cancer cells.

## 1. Introduction

Colorectal cancer (CRC) ranks as the third-commonest cancer incidence globally and the second-leading cause of cancer mortality [1]. The International Agency for Research of Cancer (IARC) under the World Health Organization (WHO) established the GLOBOCAN database to estimate incidence and mortality worldwide for 36 cancers in 185 countries. According to GLOBOCAN 2020, there are 1.93 million new cases of CRC, representing one tenth of the global cancer incidence (19.29 million new cases) [1]. CRC also accounts for 0.94 million deaths in 2020 worldwide, nearly one tenth of the total 9.96 million cancer deaths [1]. The incidence and mortality rates of CRC are highly country-specific, with wide geographical variation (more common in developed countries and gradually increasing in middle- and low-income countries due to Westernization) [2]. In Malaysia, CRC ranks second in cancer incidence and mortality in both sexes and all age groups. The gradual increase in CRC incidence and mortality rate is mainly attributed to the Westernization of lifestyle and diet and low screening awareness [3,4]. Recent advancements in medical research have led to multiple treatment options for CRC patients, including endoscopic and surgical removal, radiotherapy, chemotherapy, immunotherapy, and targeted therapy. However, due to the lack of early screening awareness, most CRC patients are already at stage III and IV during their first diagnosis, which might increase their risk of cancer relapse and poor prognosis [4,5]. Chemotherapeutics are designed to target cancer cells, but these drugs also unintentionally damage fast-growing normal cells in bone marrow, hair follicles, mouth, and gastrointestinal tract, which may lead to serious side effects, such as anemia, fatigue, massive hair loss, mouth ulcers, nausea, and diarrhea [6,7]. In addition, resistance to chemotherapy and targeted therapy is still a major challenge to improve the survival rate and prognosis of CRC patients [8,9,10]. Genetic mutations among CRC patients, such as APC, BRAF, EGFR, KRAS and PIK3CA, could influence the response to specific therapies, such as anti-EGFR and immunotherapy [11,12,13,14].

In recent years, natural compounds have been investigated to demonstrate anti-cancer properties, showing real potential for therapeutic effects in most cancers. Curcumin, also known as diferuloylmethane, is a naturally orange-yellow compound extracted from the rhizome of *Curcuma longa*, traditionally used as herbal medicine, spice, preservative, and coloring agent in Asian countries [15]. In CRC, curcumin has been reported to inhibit cell growth and induce apoptotic signaling to exert its anti-cancer potential. Curcumin induces cell cycle arrest in the human colon cancer cell line HCT116 via downregulation of cyclin D1 and CDK2 [16,17]. Curcumin also suppresses cell proliferation and induces cellular apoptosis in CRC cells via the Wnt/β-catenin signaling pathway by upregulating GSK3b and downregulating Axin2, E-cadherin, β-catenin, and c-MYC, as reviewed previously [18,19,20,21]. Curcumin also interacts with miRNAs to regulate gene expression and the downstream signaling cascade in CRC tumorigenesis. For instance, curcumin has been reported to upregulate tumor-suppressive miR-27a and miR-34a while suppressing miR-21 and miR-130a to reduce invasion and metastasis in CRC cells [22,23]. Curcumin treatment of CRC cells also downregulates miR-27a, miR-20a, and miR-17-5p to promote transcription factors ZBTB10 and ZBTB4, which inhibit several downstream target genes, including EGFR, c-MET, cyclin D1, and NFκB, resulting in cancer cell growth arrest and apoptosis induction [20,24].

Some studies suggest that curcumin synergizes with chemotherapeutics such as 5-FU, oxaliplatin and cisplatin to increase treatment efficiency and possibly overcome chemoresistance among CRC patients [25,26,27,28]. The accumulated evidence has further emphasized the potential of curcumin as a natural and multi-targeted therapeutic in CRC. However, the major drawback of using curcumin as a chemotherapeutic is its rapid metabolism and poor bioavailability [29]. Curcumin is highly hydrophobic and rapidly metabolized in the intestinal tract, resulting in limited absorption into blood circulation to reach the target site [30,31]. Several strategies have been implemented to improve the bioavailability of curcumin for systemic circulation, including the use of adjuvants, micronation, binding to cyclodextrin/liposomes, nanoformulations, and synthesis of curcumin analogues [32,33,34,35,36,37,38,39,40,41]. Among different approaches, the synthesis of novel curcumin analogues by molecular modifications has exhibited similar anti-potent effects to the original curcumin molecules but with better stability and bioavailability, which are ideal for further development into novel drugs [42]. While the basic molecular structure of curcumin comprises two phenol groups connected by two α, β unsaturated carbonyl groups, a variety of curcumin analogues have been developed by modifying functional moieties, such as biaryl rings, diketone and diene chain, onto different positions of the original curcumin structure [36,43,44,45,46,47,48,49,50,51]. With these modifications, curcumin analogues are superior to curcumin in term of pharmacokinetic properties, chemical stability and anti-cancer activities [46,52,53,54,55,56,57].

**Figure 1:**
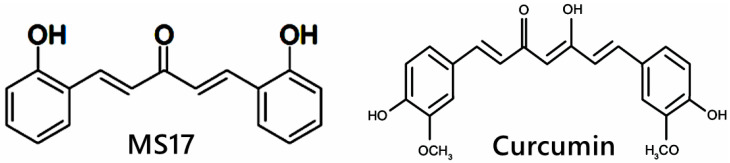
Chemical structure of MS17 and curcumin.

Diarylpentanoids (DAPs) or 1,5-diaryl-3-oxo-1,4-pentadienes are a class of monocarbonyl curcumin analogues possessing two aromatic rings linked by a five-carbon bridge, in which these DAPs are also known as C5-curcuminoids [58]. In comparison, curcumin possesses two aromatic rings linked by a seven-carbon tether that contains two α,β-unsaturated ketone moieties [59]. Multiple studies have shown that DAPs, such as CDF, EF24, GO-Y030 and UBS109, are superior to curcumin molecules in inducing growth-suppressive and apoptosis activities in a wide range of cancer cells, including breast [60], pancreatic [61], lung [62], prostate [63] and CRC [64]. For example, EF24 is a fluorinated DAP that induces apoptosis and cell cycle arrest in CRC cells via accumulation of reactive oxygen species (ROS), mitochondrial dysfunction and activation of caspase-dependent pathways [65]. CDF synergizes with 5FU and oxaliplatin to induce apoptosis in chemo-resistant CRC cells by suppressing membrane transporter ABCG2, EGFR, NF-κB, β-catenin, COX-2, c-Myc and Bcl-xL [66]. However, the research for DAPs is still relatively new, unlike curcumin studies. In addition, 1,5-bis(2-hydroxyphenyl)-1,4-pentadiene-3-one (MS17) is one of the curcumin DAPs that has been reported to exhibit anti-cancer effects on prostate cancer cells and cervical cancer cells by inducing growth inhibition and cellular apoptosis [52,67]. The structural difference between MS17 and curcumin is shown in Figure 1. To the best of our knowledge, this is the first study to investigate the anti-cancer potential of MS17 in colon cancer cells. The present study aimed to determine the cytotoxicity, anti-proliferative and apoptotic activity of 1,5-bis(2-hydroxyphenyl)-1,4-pentadiene-3-one (MS17) on human primary (SW480) and metastatic (SW620) colon cancer cells as well as to determine the underlying molecular mechanisms by identifying differentially expressed proteins (DEPs) and molecular pathways using shotgun proteomics.

## 2. Results

### 2.1. Cytotoxicity and Selective Index of MS17 and Curcumin in SW480 and SW620 Colon Cancer Cells

**Figure 2:**
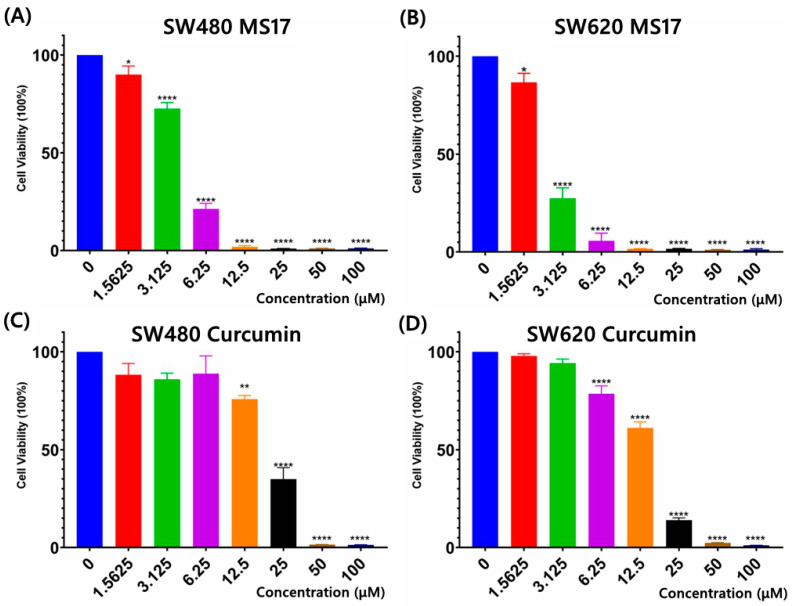
Cytotoxicity assays using different concentrations of MS17 treated on (**A**) SW480 cells and (**B**) SW620 cells and curcumin on (**C**) SW480 cells and (**D**) SW620 cells. All the experiments were performed in triplicate, and results were compared between three independent experiments. Statistically significant differences between the means of values obtained with treated vs. untreated are represented by * for *p* ≤ 0.05, ** for *p* ≤ 0.01, and **** for *p* ≤ 0.0001. Data are presented as means ± SEM.

The cytotoxicity of MS17 in SW480 and SW620 cells was evaluated using an MTT assay upon 72 h treatment. MS17 showed a significant dose-dependent cytotoxicity effect in SW480 (Figure 2A) and SW620 cells (Figure 2B). Cytotoxicity assays using curcumin were also conducted on SW480 (Figure 2C) and SW620 cells (Figure 2D) to compare against MS17. The EC_50_ values of MS17 and curcumin in SW480 and SW620 cells were calculated, as shown in Supplementary Table S1. The EC_50_ values of MS17 in SW480 and SW620 cells were 4.10 and 2.50 µM lower than the EC_50_ values of curcumin (SW480: 17.50 µM; SW620: 13.10 µM). These results also suggest that MS17 demonstrates improved cytotoxicity in SW480 and SW620 cells in a dose-dependent manner at lower concentrations than the parent compound, curcumin. Additionally, SW620 cells were more susceptible to MS17 treatment with a lower EC_50_ value than SW480 cells.

The cytotoxic effects of MS17 and curcumin were further evaluated on the non-cancerous lung fibroblast cell line WI38 (Supplementary Table S1). The EC_50_ values of both compounds were higher in WI38 (MS17: 4.9 µM; curcumin: 25.8 µM) than in cancer cell lines, indicating a higher concentration of MS17 and curcumin was required to reduce WI38 cell viability by 50%. The selective index (SX) of MS17 and curcumin was calculated by comparing EC_50_ values between respective colon cancer cells and normal fibroblasts. MS17 and curcumin showed SX values above 100 in SW480 (MS17: 119.5; curcumin: 147.4) and SW620 cells (MS17: 196; 196.9), indicating that both compounds were more selective towards cancer cells than normal cells (Supplementary Table S1).

### 2.2. Anti-Proliferative Effect of MS17 in SW480 and SW620 Colon Cancer Cells

**Figure 3:**
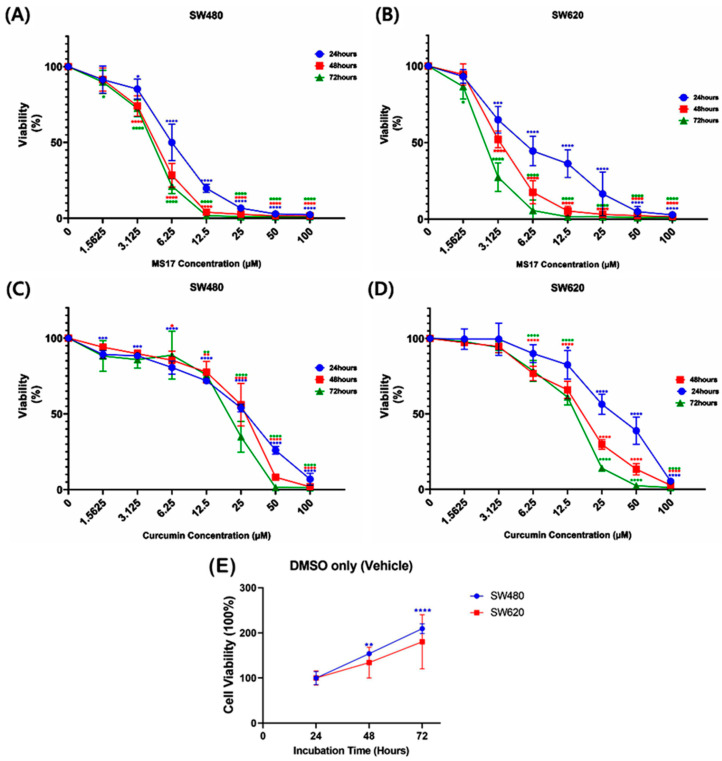
The anti-proliferative effect of MS17 in (**A**) SW480 and (**B**) SW620 cells and curcumin in (**C**) SW480 and (**D**) SW620 cells at 24, 48 and 72 h, in which (**E**) 0.2% of DMSO treatment (vehicle only) in the control wells of both cell lines did not induce anti-proliferative effects. All the experiments were performed in triplicate, and results were compared between three independent experiments. Statistically significant differences between the means of values obtained with treated vs. untreated are represented by * for *p* ≤ 0.05, ** for *p* ≤ 0.01, *** for *p* ≤ 0.001 and **** for *p* ≤ 0.0001. Data are presented as means ± SEM.

Regarding the anti-proliferative effect of MS17 in SW480 and SW620 cells for 24, 48 and 72 h, MS17 significantly reduced SW480 cell viability at 6.25 µM onwards for the 24 h incubation period and 3.13 µM onwards for 48 and 72 h (Figure 3A). Cell viability was less than 50% when treated with MS17 at 12.5 µM (19.8%) for 24 h as well as 6.25 µM for 48 h (28.47%) and 72 h (21.26%) compared to the untreated cells. MS17 also inhibited SW620 cell proliferation at a lower dosage than the SW480 cells across all the time points. A significant decline in SW620 cell proliferation was noted at 3.13 µM of MS17 treatment for all time points (24 h: 64.93%; 48 h: 52.11%; 72 h: 27.42%) compared to the untreated control (Figure 3B). MS17 treatment showed dose- and time-dependent reductions in the proliferation of SW480 and SW620 cells. MS17 treatment for 48 h and 72 h significantly induced anti-proliferative activity in SW480 and SW620 cells.

Compared to MS17, curcumin displayed a moderate anti-proliferative effect on both cancer cell lines. In SW480 cells, curcumin significantly inhibited cell viability at 1.56 µM (89.21%) upon 24 h treatment, 6.25 µM (85.45%) at 48 h and 12.5 µM (75.8%) for 72 h treatment (Figure 3C). A similar pattern of anti-proliferative effect was observed upon curcumin treatment on SW620 cells (Figure 3D). Upon 24 h treatment, 12.5 µM of curcumin significantly reduced SW620 cell viability to 82.56%, while 6.25 µM of curcumin was required to significantly reduce the cell viability of the SW620 cells to 76.8% and 78.56% at 48 and 72 h respectively.

### 2.3. Induction of Apoptosis by MS17 Treatment in SW480 and SW620 Colon Cancer Cells

The treatment conditions used for apoptotic analysis were MS17 treatment of 1× EC_50_ (SW480: 4.1 µM, SW620: 2.5 µM) and 2× EC_50_ (SW480: 8.2 µM, SW620: 5 µM). The apoptotic effect of MS17 on SW480 and SW620 cells was further evaluated with the morphological assessment using fluorescence microscopy and protein capillary electrophoresis using the Simple Western technique to investigate caspase-3 cleavage and Bcl-2 protein expression.

#### 2.3.1. Morphological Observation and Quantitative Analysis of MS17-Treated Colon Cancer Cells

The morphological analysis of MS17-treated SW480 and SW620 cells upon 24, 48 and 72 h incubation was conducted using acridine orange (AO) and propidium iodide (PI) double-staining under a fluorescent microscope. Viable cells are characterized by intact membranes with uniform green nuclei, indicating no sign of apoptosis. Early apoptotic cells exhibit bright green nuclei with distinct morphological changes, such as membrane blebbing, shrinkage of the cells and chromatin condensation. Late apoptotic cells appear as yellowish orange to bright red cells due to increased permeability of PI and condensed chromatin features. Necrotic cells exhibit uniform orange or red nuclei as these cells are fully permeable to AO and PI.

**Figure 4:**
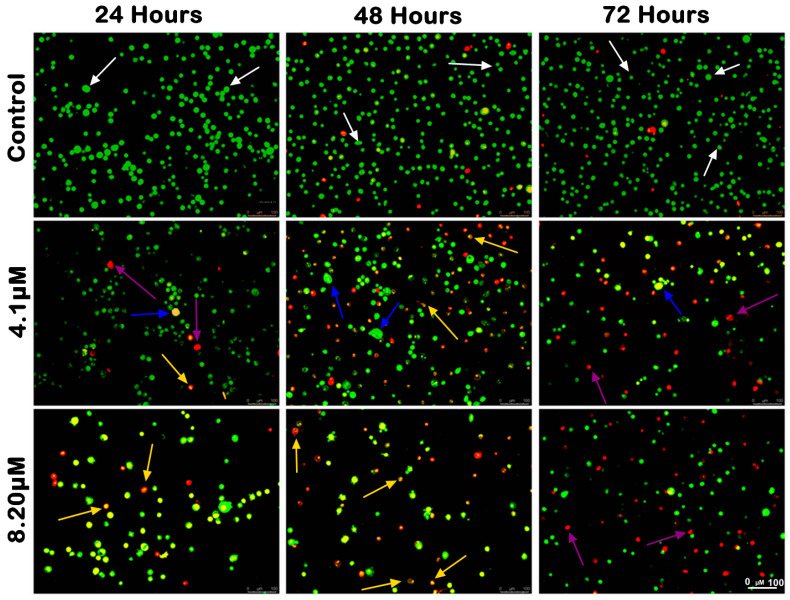
Morphological and quantitative analysis of apoptotic cells by acridine orange–propidium iodide (AO/PI) in SW480 cells upon MS17 treatment for 24, 48 and 72 h. Untreated viable cells emit uniformly green fluorescence (white arrow), while early apoptotic cells emit dense, bright-green fluorescence with membrane blebbing and chromatin condensation (blue arrow). Late apoptotic cells appear bright orange–red with yellow beads (yellow arrow). Necrotic cells showed a red appearance (purple arrow). Magnification 100×.

**Figure 5:**
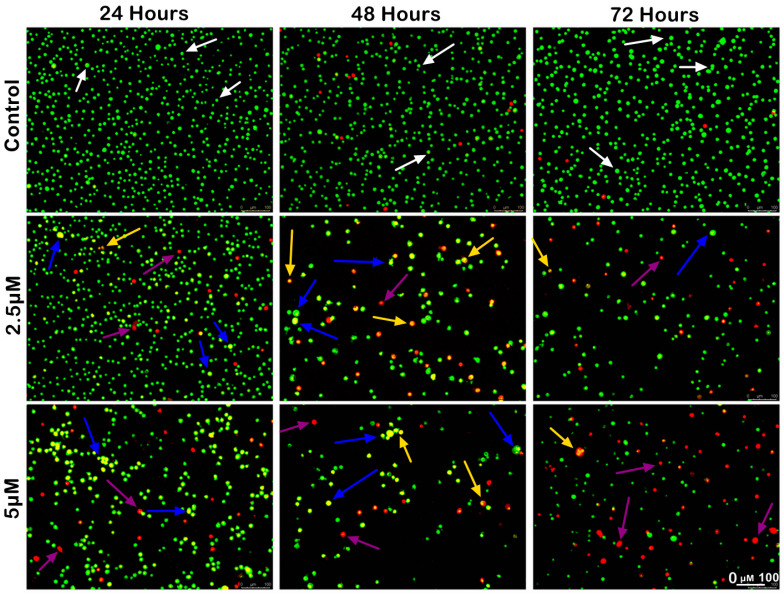
Morphological and quantitative analysis of apoptotic cells by acridine orange–propidium iodide (AO/PI) in SW620 cells upon MS17 treatment for 24, 48 and 72 h. Untreated viable cells emit uniformly green fluorescence (white arrow), while early apoptotic cells emit dense, bright-green fluorescence with membrane blebbing and chromatin condensation (blue arrow). Late apoptotic cells appear bright orange–red with yellow beads (yellow arrow). Necrotic cells showed a red appearance (purple arrow). Magnification 100×.

The morphological changes of MS17-treated SW480 and SW620 cells are shown in Figure 4 and Figure 5, respectively. The untreated control cells were mainly viable with uniform green fluorescence (white arrow). In comparison, the MS17-treated cells showed various morphological alterations with the increasing number of apoptotic and necrotic cells as MS17 concentration and treatment time increased. As shown in Figure 4, there was a small population of early apoptotic cells (blue arrow), late apoptotic cells (yellow arrow) and necrotic cells (purple arrow) SW480 cells treated with 4.1 µM of MS17 (1× EC_50_) upon 24 h treatment. However, SW480 cells treated with 8.2 µM of MS17 (2× EC_50_) showed a higher proportion of late apoptotic and necrotic cells. At 48 h incubation, both MS17 treatment groups showed an increase in early and late apoptotic and necrotic cells, while the 8.2 µM treatment group had a higher proportion of apoptotic and necrotic cells than the 4.1 µM treatment group. After 72 h treatment, the proportion of late apoptotic and necrotic cells increased more than early apoptotic cells in both MS17 treatment groups.

**Figure 6:**
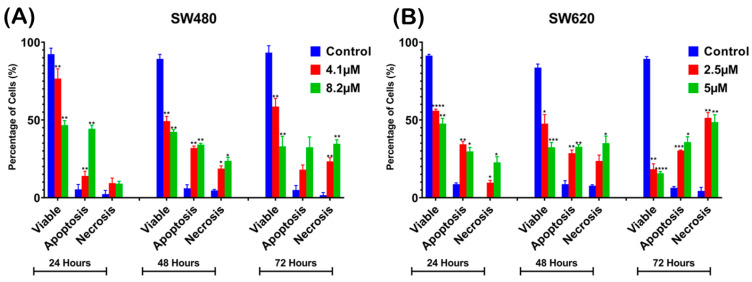
Percentage of cell population in (**A**) SW480 and (**B**) SW620 cells treated with MS17 for 24, 48, and 72 h. Results are expressed as means ± SEM from three independent experiments and comparison between data sets was performed using ANOVA. Statistically significant differences between the means of values obtained with treated vs. untreated cells are represented by * for *p* ≤ 0.05, ** for *p* ≤ 0.01, *** for *p* ≤ 0.001 and **** for *p* ≤ 0.0001.

A similar trend of apoptotic changes was observed in SW620 cells treated with 2.5 µM (1× EC_50_) and 5 µM (2× EC_50_) of MS17, respectively (Figure 4). The 2.5 µM treatment group showed few early apoptotic, late apoptotic, and necrotic cells at 24 h incubation, while the 5 µM treatment group had a higher proportion of apoptotic and necrotic cells (Figure 5). Both MS17 treatment groups had increased apoptotic and necrotic cells upon 48 h treatment, while the 5 µM treatment group had a higher proportion of apoptotic and necrotic cells. At 72 h incubation, there was a larger proportion of late apoptotic and necrotic cells in both MS17 treatment groups with fewer early apoptotic and viable cells. The percentages of apoptotic and necrotic cells are also presented in Figure 6.

For analysis purposes, early and late apoptosis were combined to indicate apoptotic activity. The percentage of viable cells decreased upon exposure to higher MS17 concentration and longer incubation times in both cell lines. In SW480 cells, the viable cells in the 8.2 µM treatment group significantly reduced to 54.33% at 24 h, 53% at 48 h and 39.67% at 72 h. As for SW620 cells, the viable cells in the 5 µM treatment group significantly reduced to 56.33% at 24 h, 48.67% at 48 h and 26.33% at 72 h.

At each time point, there was an increase in apoptotic and necrotic cells in all treatment groups compared to the untreated control. In SW480 cells, the apoptotic and necrotic cells in the 4.1 µM treatment group at 24 and 48 h were significantly increased to 9–14% and 18–32% whereas in the 8.2 µM treatment group, this was around 23–34% at 48 h. In SW620 cells, the apoptotic and necrotic cells in the 2.5 µM treatment group were significantly increased to 30 to 51% at 72 h, while the 5 µM treatment group was between 35% and 49%.

Similarly, there was an increase in the percentage of cells undergoing apoptosis and necrosis as treatment time increased in both cell lines. For instance, SW480 cells treated with 4.1 µM of MS17 showed significantly increased populations of apoptotic and necrotic cells from 9–14% at 24 h to 18–32% at 48 h. As for SW620 cells, the apoptotic and necrotic cells in the 2.5 µM treatment group significantly increased from 9–34% at 24 h to 30–51% at 72 h. Our results showed that MS17 induced time- and dose-dependent apoptotic induction in SW480 and SW620 cells.

#### 2.3.2. Quantification of Caspase-3 Activity and Bcl-2 Protein Expression upon MS17 Treatment in SW480 and SW620 Colon Cancer Cells

**Figure 7:**
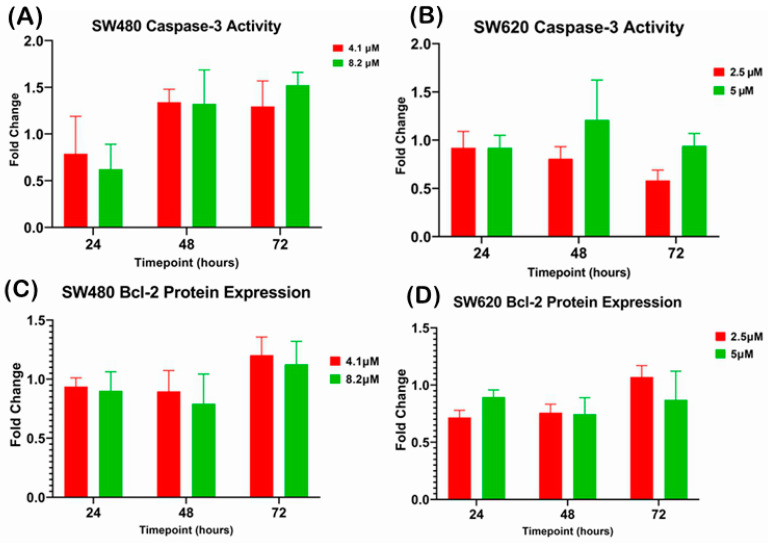
Investigation of relative caspase-3 activity in (**A**) SW480 and (**B**) SW620 cells and the Bcl-2 protein expression in (**C**) SW480 and (**D**) SW620 cells upon MS17 treatment for 24, 48 and 72 h. The fold change of protein expression in each treatment group was normalized against the untreated control. Results are expressed as means ± SEM from three independent experiments and comparison between data sets was performed using ANOVA.

There was no significant difference observed in caspase-3 activity when SW480 and SW620 cells were treated with 1 × EC_50_ (SW480: 4.1 µM; SW620: 2.5 µM) and 2 × EC_50_ (SW480: 8.2 µM; SW620: 5 µM) of MS17 at 24, 48 and 72 h (Figure 7A,B). However, SW480 cells treated with 4.1 µM and 8.20 µM of MS17 showed increased caspase-3 activity at 48 and 72 h. Similarly, no significant difference was detected for Bcl-2 protein expression in MS17-treated SW480 and SW620 cells at all three time points (Figure 7C,D).

### 2.4. Proteomic Profiling of MS17-Treated SW480 and SW620 Cells via Shotgun Proteomic Approach

Proteomic profiling of SW480 and SW620 cells upon MS17 treatment was investigated using label-free shotgun proteomic methods. The optimum dosage and treatment time selected for MS17 was 2× EC50 (SW480: 8.2 µM, SW620: 5 µM) with 24 h incubation. Based on anti-proliferation assays and morphological analysis, these parameters were selected to confirm that the cells underwent apoptosis with 50% cell viability upon exposure to 2× EC50 of MS17 for 24 h. In SW480 cells, 3048 and 1822 proteins were identified in the untreated control and the MS17 treatment group, respectively. Differentially expressed proteins (DEPs) were selected with a fold change of more than 1.10 (upregulated) or less than 0.90 (downregulated) in MS17-treated cells. A total of 24 DEPs were upregulated in the MS17-treated SW480 cells, with no downregulated DEPs. On the other hand, proteomic profiling of SW620 cells revealed that 1752 and 2295 proteins were identified in the untreated control group and the MS17 treatment group, respectively. Among these proteins, a total of 92 DEPs (66 upregulated and 26 downregulated) were identified in MS17-treated SW620 cells.

#### 2.4.1. Protein Identification and Classification

The identified DEPs in SW480 and SW620 cells were classified into respective protein classes according to PANTHER classification. There were four DEPs commonly found in MS17-treated SW480 and SW620 cells, including cytoplasmic actin (ACTB), heat shock protein HSP70 family members 1A and 1B (HSPA1A and HSPA1B), and ribosomal protein L7a (RPL7A). HSPA1A and HSPA1B were upregulated in MS17-treated SW480 and SW620 cells, while ACTB and RPL7A were upregulated in SW480 cells and downregulated in SW620 cells. The 24 DEPs in MS17-treated SW480 cells were classified into nine protein classes, as shown in Supplementary Table S2. The largest protein class was the chaperones, comprising 36.4% (8/22) of the DEPs, followed by the ribosomal proteins (13.6%, 3/22). Chromatin-regulatory proteins, metabolic enzymes, transport proteins and ubiquitin–protein ligases equally comprised 9.1% (2/22) of the DEPs, while calcium signaling protein, cytoskeletal protein and nuclear membrane protein had 1 DEP (4.5%), respectively.

The 92 DEPs in MS17-treated SW620 cells were classified into 13 protein classes, as demonstrated in Supplementary Table S3. Ribosomal proteins and RNA-binding proteins represented the largest protein classes that equally comprised 19.6% (18/92) of the DEPs, followed by the chaperones (15.2%, 14/92 DEPs). Cytoskeletal proteins and metabolic enzymes were the third-largest protein classes, each comprising 10.9% (10/92) of the DEPs, followed by calcium signaling proteins (5.4%, 5/92 DEPs). DNA-binding proteins and transport proteins constituted 4.3% (4/92) of the DEPs, while chromatin-regulatory proteins, nuclear proteins, peripheral membrane proteins, and ubiquitin–protein ligases comprised 2.2% (2/92) of the DEPs. The only scaffold protein upregulated in MS17-treated SW620 cells was RANBP1. The DEPs in MS17-treated SW480 and SW620 cells were further analyzed for gene ontology (GO) functional annotation, protein–protein interaction (PPI) network, and Reactome pathway analysis.

#### 2.4.2. Gene Ontology: Functional Annotations

**Figure 8:**
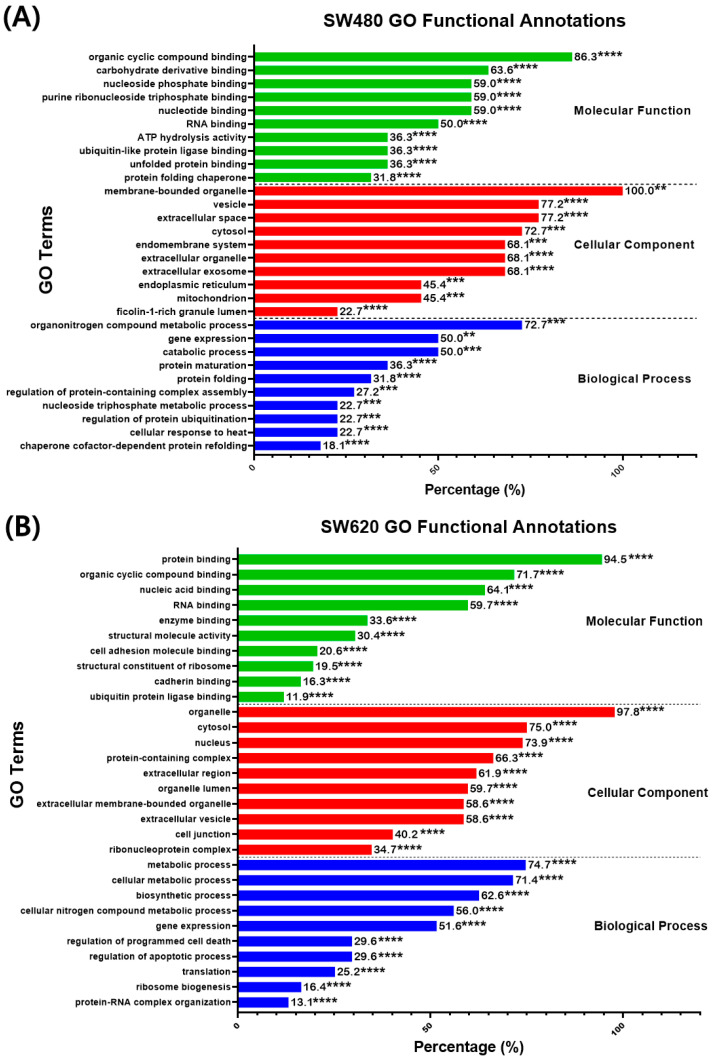
Percentage (%) of DEPs identified in MS17-treated (**A**) SW480 and (**B**) SW620 cells annotated to each statistically enriched GO term for functional categories “Molecular function”, “Cellular component” and “Biological process”. The number above each bar represents the percentage of protein of the respective GO terms, while the asterisks indicate the false-discovery rate (FDR) of each GO term upon multiple correction statistical tests using the Benjamini–Hochberg procedure (** *p* < 0.01, *** *p* < 0.001 and **** *p* < 0.0001).

The DEPs in SW480 and SW620 cells were analyzed for their gene ontology (GO) functional annotation to identify the enriched GO terms in the biological process, molecular function, and cellular component. The search results for enriched GO terms were filtered based on the FDR < 0.05 cutoff. The 10 most significant terms were selected for every GO component, as shown in Figure 8. In SW480 cells, the highly significant (*p* < 0.01, percentage ≥ 50%) GO terms in the biological process were “Organonitrogen compound metabolic process” (72.70%), “Gene expression” (50%) and “Catabolic process” (50%) as reported in Figure 8A. As for the cellular components, the most significant (*p* < 0.001) terms were “Membrane-bounded organelle” (100%), “Vesicles” (77.20%), “Extracellular space” (77.20%), “Cytosol” (72.70%), “Extracellular organelle” (68.10%), “Extracellular exosome” (68.10%), and “Endomembrane system” (68.10%). Additionally, the most significant (*p* < 0.001) enriched GO terms in the molecular function were “Organic cyclic compound binding” (86.30%), “Carbohydrate derivative binding” (63.60%), “Nucleotide-binding” (59%), “Purine ribonucleoside triphosphate binding” (59%), “Nucleoside phosphate binding” (59%), and “RNA binding” (50%).

Meanwhile, in SW620 cells, the most significant (*p* < 0.0001) GO terms in the biological process were “Metabolic process” (74.70%), “Cellular metabolic process” (71.40%), “Biosynthetic process” (62.60%), “Cellular nitrogen compound metabolic process” (56%), “Gene expression” (51.60%), as demonstrated in Figure 8B. As for the cellular components, the most significant (*p* < 0.0001) GO terms were “Organelle” (97.80%), “Cytosol” (75%), “Nucleus” (73.90%), “Protein-containing complex” (66.30%), “Extracellular region” (61.90%), “Organelle lumen” (59.70%), “Extracellular membrane-bounded organelle” (58.60%) and “Extracellular vesicle” (58.60%). Moreover, in the molecular function, the most significant (*p* < 0.0001) GO terms were “Protein binding” (94.50%), “Organic cyclic compound binding” (71.70%), “Nucleic acid binding” (64.10%) and “RNA binding” (59.70%).

#### 2.4.3. Protein–Protein Interaction (PPI) Network

Protein–protein interaction (PPI) was assessed to determine the functional interactions of DEPs in MS17-treated SW480 and SW620 cells, respectively. The interactions between DEPs were derived at a high confidence level greater than 0.7 to populate the protein groups with similar correlations. In a PPI network, nodes represent the DEPs, and edges refer to the distance between DEPs. The line thickness indicates the degree of confidence in the prediction of the connection. A total of 29 nodes and 69 edges were identified in the PPI network of DEPs in MS17-treated SW480 cells. Due to the limited number of 22 DEPs in SW480 cells, additional first-shell protein interactors directly connecting with the DEPs were included in the PPI network analysis. Among the 29 nodes, 19 DEPs were identified, including ABCA13, ACTB, HMGB1, HMGB1P1, HSP90AA1, HSPA1A, HSPA1B, HSPA5, HSPE1, NAV3, PKLR, PKM, RAN, RPL7A, RPS27A, S100A6, SERPINH1, UBA52, UBB, and UBC. The other 10 first-shell interactors in the PPI analysis were AHSA1, CDC37, FKBP4, NUTF2, PFN1, RPL18A, RPL19, RPS12, STUB1 and USP5. The DEPs in SW480 cells and those first-shell interactors demonstrated significant interactions, as indicated by the average local clustering coefficient of 0.64 (*p* < 4.72 × 10^−8^).

**Figure 9:**
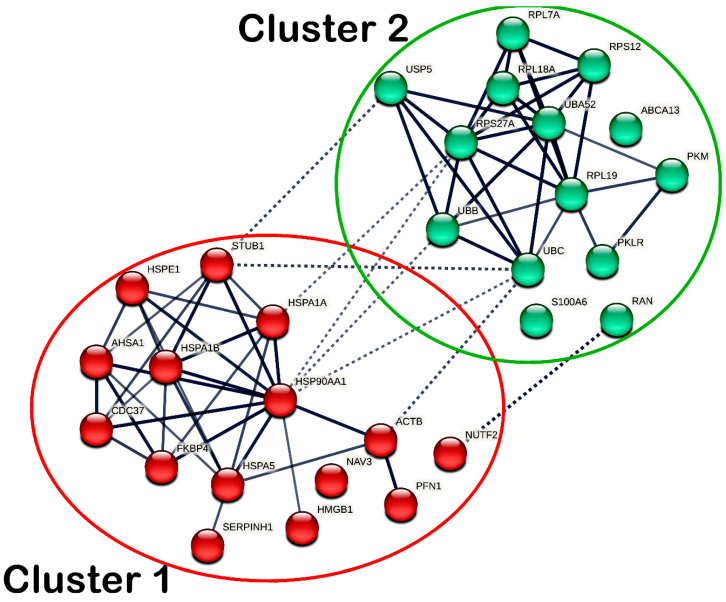
Protein–protein interaction (PPI) network of all differentially expressed proteins (DEPs) identified in MS17-treated SW480. The nodes represent proteins, wherein the nodes in the same cluster are demonstrated by the same color. The lines connecting the nodes indicate the association between the proteins. The thicker the line, the higher the confidence in the interaction prediction. Dashed lines represent inter-clusters between the highly connected clusters.

The DEPs with similar functional roles were grouped into clusters based on k-means clustering analysis, in which intra- and inter-cluster interactions were reported. As for SW480 cells, the DEPs were grouped into two different clusters, as shown in Figure 9, while the members of each cluster are listed accordingly in Supplementary Table S4. In cluster 1, the chaperones (HSP90AA1, HSPA1A, HSPA1B, HSPA5, HSPE1, and SERPINH1), ACTB, AHSA1, CDC37, FKBP4, HMGB1, PFN1, and STUB1 showed intracluster interactions. Meanwhile, in cluster 2, transport protein ABCA13, metabolic enzymes (PKLR and PKM), ribosomal proteins (RPL7A, RPL18A, RPL19, RPS12, RPS27A, UBA52) and ubiquitin–protein ligases (UBB and UBC) showed intracluster interactions. Additionally, ACTB, HSP90AA1, HSPA1A and STUB1 in cluster 1 and NUTF2, RAN, RPS27A, UBB, UBC and USP5 in cluster 2 demonstrated intercluster interactions.

**Figure 10:**
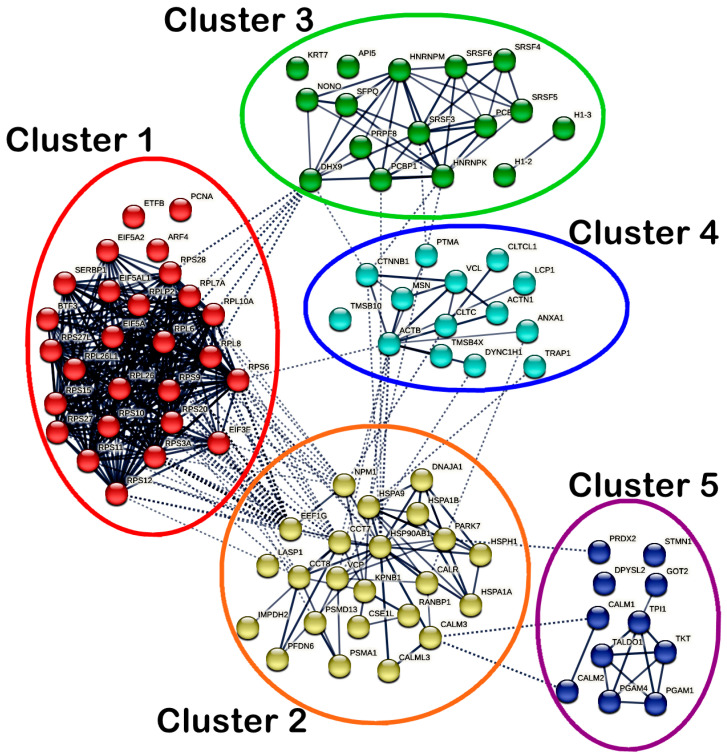
Protein–protein interaction (PPI) network of all differentially expressed proteins (DEPs) identified in MS17-treated SW620. The nodes represent proteins, wherein the nodes in the same cluster are demonstrated by the same color. The lines connecting the nodes indicate the association between the proteins. The thicker the line, the higher the confidence in the interaction prediction. Dashed lines represent inter-clusters between the highly connected clusters.

On the other hand, a total of 91 nodes and 425 edges were identified in the PPI network of MS17-treated SW620 cells. Significant interactions between the DEPs in SW620 cells were indicated with an average local clustering of 0.663 (*p* < 1 × 10^−16^). As for SW620 cells, a total of five highly connected clusters were generated, as shown in Figure 10, while members of each cluster are listed accordingly in Supplementary Table S5. Cluster 1 comprised ribosomal proteins (RPL10A, RPL26, RPL26L1, RPL6, RPL7A, RPL8, RPLP2, RPS10, RPS11, RPS12, RPS15, RPS20, RPS27, RPS27L, RPS28, RPS3A, RPS6, RPS9), RNA-binding proteins (EIF3F, EIF5A, EIF5A2, EIF5AL1, SERBP1) and DNA-binding protein BTF3 and showed dense intracluster and intercluster interactions. Cluster 2 consisted of chaperones (CALR, CCT7, CCT8, DNAJA1, HSP90AB1, HSPA1A, HSPA1B, HSPA9, HSPH1, NPM1, PARK7, PFDN6), transport proteins (CSE1L, KPNB1, VCP), ubiquitin–protein ligases (PSMA1, PSMD13), calcium signaling proteins (CALM3 and CALML3), scaffold protein RANBP1, RNA-binding protein EEF1G, metabolic enzyme IMPDH2 and cytoskeletal protein LASP1 and showed strong intracluster interactions. In addition, several DEPs in cluster 2 (CALML3, CALR, CCT7, CCT8, EEF1G, HSP90AB1, HSPA1B, HSPA9, NPM1, PARK7 and VCP) also showed multiple intercluster interactions. Clusters 1 and 2 also showed greater intercluster interactions with each other compared to the interactions with other clusters.

Cluster 3 mainly comprised RNA-binding proteins (DHX9, HNRNPK, HNRNPM, NONO, PCBP1, PCBP2, PRPF8, SFPQ, SRSF3, SRSF4, SRSF5 and SRSF6) and showed intracluster interactions. Moreover, DHX9 also showed intercluster interactions with RPL6, RPL7A, RPL8, RPL10A and RPS28 in cluster 1 and CTNNB1 in cluster 4, while HNRNPK and SRSF3 also interacted with PTMA in cluster 4. PCBP1 in cluster 3 also interacted with HSP90AB1 in cluster 2. As for cluster 4, intracluster interactions existed between cytoskeletal proteins (ACTB, ACTN1, LCP1, MSN and TMSB4X), DNA-binding proteins (CTNNB1, DYNC1H1), peripheral membrane proteins (CLTC, CLTCL1), calcium signaling protein (ANXA1), nuclear protein (PTMA), transport protein (VCL). Notably, ACTB, ANXA1, CLTC, CTNNB1, DYNC1H1 and TRAP1 in cluster 4 showed intercluster interactions with several DEPs (CALR, CCT7, HSP90AB1, HSPA9, NPM1 and VCP) in cluster 2. CTNNB1 interacted with HNRNPK in cluster 3. In cluster 5, the metabolic enzymes (GOT2, PGAM1, PGAM4, TALDO1, TKT and TPI1) and calcium signaling proteins (CALM1 and CALM2) showed intracluster interactions with the same protein classes, respectively. Metabolic enzyme PRDX2 in cluster 5 showed intercluster interaction only with PARK7 in cluster 2, while CALM1 and CALM2 also interacted with CALM3 in cluster 2.

#### 2.4.4. Reactome Pathway Analysis

All DEPs identified in MS17-treated SW480 and SW620 cells were mapped into the Reactome Pathway Database to identify the molecular pathways modulated by MS17. An overrepresentation analysis was performed. “Cellular responses to stress” was commonly identified in SW480 and SW620 cells. The rest of the most enriched pathways identified in SW480 cells were “Regulation of PTEN localization,” “Downregulation of ERBB4 signaling,” “Signaling by EGFR in cancer,” “Downregulation of ERBB2 signaling,” and “Signaling by NOTCH1 HD domain mutants in cancer.” Meanwhile, for SW620 cells, the top enriched pathways were “Nonsense-mediated decay (NMD),” “Eukaryotic translation elongation,” “Regulation of expression of SLITs and ROBOs,” “Metabolism of amino acids and derivatives,” and “Gene and protein expression by JAK-STAT signaling after interleukin-12 stimulation.” The DEPs mapped to each pathway are shown in Table 1 for SW480 cells and Table 2 for SW620 cells.

## 3. Discussion

The present study showed that MS17 (1,5-bis(2-hydroxyphenyl)-1,4-pentadiene-3-one) inhibited SW480 and SW620 colon cancer cell growth, which was shown to be more cytotoxic than curcumin in both cell lines. Based on the cytotoxicity assay, the EC_50_ values of MS17 (SW480: 4.1 µM; SW620: 2.5 µM) were four- to fivefold lower than curcumin (SW480: 17.50 µM; SW620: 13.10 µM). MS17 also induced cytotoxic effects in SW480 and SW620 cells in a dose-dependent manner. Our results support findings in previous studies, in which several DAPs, such as EF24, GO-Y030 and MS13, were more cytotoxic than curcumin in CRC cells [64,65,68]. Previously, our group has reported the enhanced cytotoxicity of MS17 in breast, cervical, prostate, and lung cancer cells [52,67,69]. In the present study, MS17 demonstrated enhanced cytotoxicity compared to curcumin treatment in SW480 and SW620 colon cancer cells, which could be attributed to the structural modification of the curcumin molecule. MS17 was synthesized by removing β-diketone from the seven-carbon backbone and substituting a hydroxy group on each phenyl ring [70]. These modifications contributed towards the chemical stability of MS17, resulting in higher cytotoxicity and enhanced anti-proliferative effect than curcumin [71,72,73]. In addition, SX values of MS17 in SW480 and SW620 cells were above 100, suggesting that MS17 is more selective towards colon cancer cells than normal cells, which is ideal for further development into cancer therapy.

The anti-proliferation assay also showed that MS17 significantly reduced SW480 and SW620 cell viability at lower concentrations than curcumin at all three time points (24, 48 and 72 h). For instance, upon 24 h incubation, MS17 treatment of 6.25 µM significantly reduced SW480 and SW620 cell viability to about 50%, while 25 µM of curcumin was required to achieve similar cytotoxicity. Our study is the first to report on the anti-proliferative effect of MS17 in primary SW480 and metastatic SW620 colon cancer cells. MS17 treatment of 3.13 µM significantly reduced the cell viability of SW480 and SW620 cells upon 24 h incubation, and the effect became more prominent with increasing treatment period and compound concentration. Therefore, MS17 induced anti-proliferative effect in SW480 and SW620 cells in dose- and time-dependent manners. Additionally, our results demonstrated that MS17 had a lower EC_50_ value in SW620 cells than the primary SW480 cells. MS17 also induced a stronger anti-proliferative effect in SW620 cells than in SW480 cells. These findings suggest that the SW620 metastatic colon cancer cells could be more susceptible to cytotoxic and anti-proliferative effects of MS17 compared to the SW480 primary colon cancer cells. The SW480 and SW620 cells were derived from the same patient at different stages of tumor development: SW480 from a primary adenocarcinoma of the colon and SW620 from a lymph node metastasis [74,75]. Several studies have highlighted the differences in biological characteristics and molecular features between these two cell lines, which are essential for studying the molecular mechanisms and genetic alterations in CRC progression [75,76,77,78,79,80,81,82,83,84]. Previous studies showed that SW620 cells were more sensitive to curcumin than SW480 cells [85,86]. Isnida et al. also reported that the EC_50_ value of MS13 was lower in SW620 cells than in SW480 cells [64]. The present findings suggest that the biological variations between SW480 and SW620 cells may affect the treatment response towards MS17, with higher cytotoxicity against SW620 metastatic colon cancer cells.

Apoptosis plays a crucial role in cancer progression, where dysregulation in the apoptotic pathways leads to the malignant transformation of cancer cells, tumor metastasis and treatment resistance. Anti-cancer compounds can potentially target the apoptotic mechanisms in cancer cells to induce treatment effects. In the present study, the apoptotic effects of MS17 in SW480 and SW620 cells were investigated using morphological analysis to visualize the apoptotic changes in treated cells and assay kits to measure the caspase-3 activity and Bcl-2 protein expression. As shown in the morphological analysis, MS17 treatment of 1× EC_50_ (SW480: 4.10 µM, SW620: 2.5 µM) induced early apoptotic changes, such as membrane blebbing, chromatin condensation, nuclear fragmentation, and cell shrinkage, in SW480 and SW620 cells upon 24 h incubation. As the MS17 concentration increased to 2× EC_50_ (SW480: 5 µM, SW620: 8.20 µM) while the treatment time extended to 48 and 72 h, the population of apoptotic and necrotic cells also significantly increased in both cell lines, with significant reduction of viable cells as we presented in the quantitative analysis. Our results demonstrated that MS17 significantly induced dose- and time-dependent apoptotic effects in SW480 and SW620 cells.

Caspase-3 and Bcl-2 are two important proteins involved in the regulation of apoptosis via intrinsic pathways [87]. Caspase-3 is an enzyme that executes the final steps of apoptosis by cleaving various cellular substrates, including cytoskeletal proteins, nuclear proteins, and DNA repair enzymes [87]. Bcl-2 is a protein that inhibits apoptosis by preventing the release of cytochrome C from the mitochondria, which triggers caspase activation [87]. Activation of caspase-3 can lead to the morphological changes associated with apoptosis and DNA damage, such as chromatin condensation, cell shrinkage and membrane distortion [88,89,90,91]. In the present study, there were no significant results on caspase-3 activity and Bcl-2 reduction in SW480 and SW620 cells upon MS17 treatment for 24 to 72 h. Previous studies from our group showed that MS17 significantly promoted caspase-3 activity in cervical and prostate cancer cells, associated with the apoptotic changes in cell morphology [52,67]. However, there are no previous data regarding the effect of MS17 on Bcl-2 protein expression in cancer cells. In addition, curcumin has been reported to induce apoptosis in CRC cells via the upregulation of caspase-3 and downregulation of Bcl-2 protein [92,93,94,95]. Numerous curcumin analogues have been reported to promote apoptosis in cancer cells by inducing caspase-3 cleavage/activity and Bcl-2 protein suppression [63,96,97,98,99]. For instance, our group also reported another curcumin analogue MS13 significantly promoted caspase-3 activity and downregulated Bcl-2 protein expression in SW480 and SW620 cells [64]. Based on our findings, MS17 can induce alternative apoptotic mechanisms in SW480 and SW620 cells, which are independent of caspase-3 activity and Bcl-2 suppression. Further investigation is essential.

Proteomics analysis is a powerful approach to provide insights into the molecular mechanisms of biological systems and processes. The present study utilized mass spectrometry to identify the differentially expressed proteins (DEPs) in SW480 and SW620 cells upon MS17 treatment of 2× EC_50_ (SW480: 8.20 µM, SW620: 5 µM) for 24 h. The proteomic profiling identified 24 upregulated DEPs in MS17-treated SW480 cells, while SW620 cells showed 66 upregulated DEPs and 26 downregulated DEPs. These DEPs were classified into respective protein classes, of which the top 2 in SW480 were chaperones and ribosomal proteins. The top three protein classes in SW620 cells were ribosomal proteins, RNA-binding proteins, and chaperones. MS17 mainly targeted ribosomal proteins and chaperones in SW480 and SW620 cells, suggesting that these protein classes could be associated with MS17-induced apoptosis in colon cancer cells. Additionally, MS17 also targeted several top protein classes in SW620 cells, including RNA-binding proteins, cytoskeletal proteins, and metabolic enzymes, which implies that MS17 could target gene transcription and translation, cellular integrity, and cancer metabolism to induce anti-cancer effects, such as growth inhibition, anti-proliferation, and apoptosis.

As for GO enrichment analysis, the most common and highly significant GO terms associated with the DEPs in MS17-treated SW480 cells were “Organonitrogen compound metabolic process” in the biological process, “Membrane-bounded organelle” in the cellular component and “Organic cyclic compound binding” in the molecular function. As for SW620 cells, the most common and highly significant GO terms were “Metabolic process” in the biological process, “Organelle” in the cellular component, and “Protein binding” in the molecular function. In addition, several highly significant GO terms were commonly reported in SW480 and SW620 cells, including “Gene expression” in the biological process, as well as “Organic cyclic compound binding” and “RNA binding” in the molecular function. Our analysis showed that MS17 can demonstrate anti-cancer effects in SW480 and SW620 cells by targeting gene expression, metabolic process, organic cyclic compound binding, protein binding and RNA binding.

Protein–protein interaction network (PPIN) analysis provides insights into the molecular networks of protein complexes that enable the cells to function [100,101]. In the present study, PPIN analysis of all DEPs was constructed using the STRING database and similar functional proteins were grouped into clusters using the k-means clustering approach [102]. The 19 DEPs in MS17-treated SW480 cells were grouped into two clusters with the addition of 10 first-shell protein interactors to complete the interaction networks and enhance the overall analysis. These two protein clusters in SW480 cells demonstrated strong intra- and inter-cluster interactions. Similarly, the 91 DEPs in SW620 cells were grouped into five protein clusters that showed dense intra- and inter-cluster interactions. MS17 may induce anti-cancer effects in SW480 and SW620 cells by modulating the intra- and inter-cluster interactions between the DEPs. By integrating the PPI network analysis into the Reactome pathway analysis, our findings demonstrated that these protein interactions were significantly associated with the molecular pathways modulated by MS17 in SW480 and SW620 cells. In MS17-treated SW480 cells, chaperones in cluster 1 (HSP90AA1, HSPA5, HSPA1A, HSPA1B) showed intercluster interactions with ribosomal proteins (RPL7A, RPS27A, UBA52) and ubiquitin–protein ligases (UBB, UBC) in cluster 2, which were mapped into “Cellular response to stress” as the most significantly associated pathway. The intercluster interaction between HSP90AA1 in cluster 1 and RPS27A, UBA52, UBB and UBC in cluster 2 were also significantly associated with “Signaling by EGFR in cancer” and “Downregulation of ERBB2 signaling.” The intracluster interactions between RPS27A, UBA52, UBB and UBC were also commonly associated with the abovementioned pathways, “Downregulation of ERBB4 signaling,” “Regulation of PTEN localization” and “Signaling by NOTCH1 HD domain mutants in cancer.”

Cellular stress response refers to a wide range of molecular changes that cells undergo in response to environmental stressors, including temperature fluctuation, exposure to toxins, viral infections and mechanical damage [103]. Heat shock proteins (HSPs) are the essential regulators of cellular stress response by acting as chaperones to assist proper folding and assembly of other proteins [104]. HSP protein families are classified based on molecular weights, such as HSP40, HSP60, HSP70 and HSP90. Our data showed that several members of the HSP70 family (HSPA5, HSPA1A, and HSPA1B) and HSP90 protein (HSP90AA1) were upregulated in MS17-treated SW480 cells. HSPA1A and HSPA1B are the stress-inducible forms of the HSP70 family, while HSPA5 is mainly located in the endoplasmic reticulum (ER) lumen to facilitate ER homeostasis and regulate cell metabolism [105]. HSP70 and HSP90 work together to fold and remodel cellular proteins by forming a multi-chaperone complex, a critical component in protein homeostasis and cellular processes [106]. Multiple studies have reported that curcumin can induce HSP70 upregulation in different cancer cells, including CRC, cervical, leukemia and lung cancer cells, mainly associated with reactive oxygen species (ROS) formation and apoptotic induction [107,108,109,110,111]. Curcumin analogues C509, C521, and C524 have been shown to upregulate HSPA5 and stress-related unfolded protein response (UPR) genes (ATF4, XBP1, and DDIT3) in the pancreatic cancer cells, which could be responsible for inducing mitochondrial membrane depolarization, caspase-3 activation and DNA breakdown, leading to apoptosis [112]. Our data suggest that MS17-upregulated HSP70 and HSP90 proteins could be associated with cytotoxicity, anti-proliferation, and apoptosis in SW480 cells, which merits further investigation on the underlying mechanisms.

Meanwhile, upregulated HSP90AA1 in MS17-treated SW480 cells was also significantly associated with “Signaling by EGFR in cancer” and “Downregulation of ERBB2 signaling.” EGFR (epidermal growth factor receptor), also known as ErbB1/HER1, is a receptor tyrosine kinase that belongs to the ErbB family [113]. EGFR signaling is among the most critical pathways in cancers, which promotes proliferation, cell cycle progression, invasion and metastasis [114]. ERBB2 is another member of the ErbB family that regulates cell proliferation, migration, differentiation, apoptosis, and cell motility by activating downstream signaling pathways, such as AKT, MAPK, and PI3K [115]. Overexpression of ERBB2 due to gene amplification or mutations has been reported in about 2% to 5% of metastatic CRC and is associated with resistance to anti-EGFR therapy [116,117]. Previously, HSP90 has been shown to restrain the heterodimerization of ERBB2 and prevent its activation [118]. Our findings suggest that MS17 may downregulate EGFR and ERBB2 signaling in SW480 cells via the upregulation of HSP90AA1, which could be beneficial to improve treatment response towards targeted therapy. Furthermore, ERBB4 is another member of the ErbB family involved in the regulation of cell growth and differentiation [119]. ERBB4 is overexpressed in human colon cancer associated with enhanced cellular transformation [120]. ERBB4 has been reported to protect colon epithelial cells from TNF-induced apoptosis and promote their survival and growth [121]. ERBB4 may also interact with other members of the ErbB family, such as EGFR and ERBB2, to modulate their signaling and response to targeted therapies [113,119]. In the present study, the upregulation of RPS27A, UBA52, UBB and UBC in MS17-treated SW480 cells may have promoted the downregulation of ERBB4 signaling, leading to growth inhibition and apoptosis.

Notch signaling is a major pathway involved in intercellular interaction and cell differentiation, and also plays a vital role in the proliferation, apoptosis, differentiation, invasion and metastasis of cancer cells [122]. In CRC, Notch signaling regulates cancer stem cell (CSC) features, and mutations in Notch signaling are associated with enhanced anti-cancer effects andimmune response [123,124,125]. Curcumin can induce cell cycle arrest and apoptosis by inhibiting the Notch signaling pathway in cancer cells [126,127,128,129,130]. The curcumin analogue GO-Y030 is another diarylpentanoid reported to inhibit Notch signaling, STAT3 phosphorylation and tumor sphere formation in colon cancer stem cells, resulting in apoptosis [68]. Furthermore, phosphatase and tensin homologue (PTEN) is a crucial tumor suppressor that inhibits cell proliferation and induces apoptosis by suppressing PI3K-Akt and other growth signaling pathways [131,132]. PTEN is frequently inactivated in cancers, but it has been reported with mutations in about 8 to 10% of CRC cases, which are highly associated with cancer metastasis, advanced tumor staging and poor prognosis [133]. Difluorinated-curcumin (CDF) is a curcumin analogue that can modulate PTEN expression in CRC and pancreatic cancer cells [134,135,136]. Sanchita et al. reported that CDF suppressed miR-21 in chemo-resistant colon cancer cells to restore PTEN expression and downregulate Akt signaling, which inhibited cell proliferation [135].

Our analysis showed that the upregulation of RPL7A, UBA52, UBB and UBC was commonly associated with all the top significant pathways in MS17-treated SW480 cells, suggesting the importance of these DEPs underlying the molecular mechanisms of MS17 to target signaling pathways and induce anti-cancer effects in colon cancer cells. Ribosomal proteins RPL7A and UBA52 are part of the large 60S ribosomal subunit, while RPS27A is part of the small 40S ribosomal subunit [137,138]. UBA52 and RPS27A are the fusion proteins formed with a single copy of ubiquitin fused to the ribosomal proteins RPL40 and RPS31, respectively [139,140,141]. UBB and UBC are polyubiquitin precursor proteins cleaved by specific proteases to generate free ubiquitin molecules in proteasome-mediated proteolysis [142]. RPS27A, UBA52, UBB and UBC play essential roles in the ubiquitin–proteasome system to regulate protein stability and degradation in nearly all cellular processes [143,144,145,146,147,148,149]. Our findings suggest that MS17 may promote RPS27A, UBA52, UBB and UBC in SW480 cells to facilitate protein ubiquitination and degradation of essential components involved in multiple signaling pathways, including EGFR, ERBB2, ERBB4, Notch and PTEN, which could be associated with growth inhibition, anti-proliferation and apoptosis.

On the other hand, “Cellular responses to stress” in MS17-treated SW620 cells was significantly associated with ribosomal proteins in cluster 1 (RPS9, RPS6, RPS27L, RPS3A, RPL10A, RPL8, RPL6, RPS15, RPL7A, RPS28, RPS27, RPLP2, RPS20, RPL26, RPS11, RPS10, RPL26L1, RPS12), chaperones in cluster 2 (CALR, DNAJA1, HSP90AB1, HSPA1A, HSPA1B, HSPA9 and HSPH1), transport protein VCP in cluster 2, ubiquitin–protein ligases in cluster 2 (PSMA1, PSMD13), histones in cluster 3 (H1-2, H1-3), cytoskeletal protein MSN and DNA binding protein DYNC1H1 in cluster 4 and metabolic enzymes in cluster 5 (PRDX2, TALDO1, TKT), which have demonstrated multiple intercluster interactions. Among these DEPs, ribosomal protein RPL7A and chaperones HSPA1A and HSPA1B were commonly associated with cellular stress response in SW480 and SW620 cells, in which HSPA1A and HSPA1B were mutually upregulated in both cell lines, but RPL7A was upregulated in SW480 cells and downregulated in SW620 cells. Other ribosomal proteins, such as RPL10A, RPL8, RPL6, RPLP2, RPL26, and RPL26L1, are part of the large 60S ribosomal subunit, while RPS9, RPS6, RPS27L, RPS3A, RPS15, RPS28, RPS27, RPS20, RPS11, RPS10 and RPS12 belong to the small 40S ribosomal subunit. RPLP2, RPL7A, RPS10, and RPS28 were significantly downregulated, while the remaining ribosomal proteins were upregulated in MS17-treated SW620 cells.

Previous studies have shown that several ribosomal proteins are overexpressed in CRC, including RPL6, RPL8, RPS3A, RPS6, RPS11, RPS12 and RPS27, but their functional importance is still unknown [150,151,152,153,154]. Accumulating evidence suggests that the extra-ribosomal functions of ribosomal proteins in CRC cells could be essential to facilitate tumorigenesis. For instance, the knockdown of RPS9 in HT29 colon cancer cells induces growth inhibition and cell cycle arrest at the G2/M phase by downregulating the cell cycle regulator CDK1 [155]. Overexpression of RPS27L in LoVo colon cancer cells enhances DNA repair capacity and inhibits apoptosis [156]. Germline mutation of RPS20 is regarded as a rare cause of hereditary non-polyposis CRC [157,158]. Increased expression of RPS28 in sporadic CRC is associated with tumor progression, which could become a potential biomarker [159]. In addition, several ribosomal proteins in our study (RPL26, RPS15, RPS20, RPS27 and RPS27L) have been reported to regulate the p53 pathway upon activation by DNA damage, oxidative stress and other stimuli [160]. These ribosomal proteins can bind to MDM2, an E3 ubiquitin ligase that targets p53 for degradation, inhibiting its activity that subsequently leads to the stabilization and activation of p53, resulting in cell cycle arrest and apoptosis [160]. Researchers have proposed that ribosomal proteins could function as the crucial regulators of the p53-mediated stress response in cancer cells [160]. Our findings showed that MS17 regulated the expression of multiple ribosomal proteins in SW620 cells, which could be associated with the extra-ribosomal functions of these DEPs to induce apoptotic effects by affecting the cellular responses to stress.

Several chaperones, including the upregulated DEPs (DNAJA1, HSP90AB1, HSPA1A, HSPA1B and HSPH1) and the downregulated DEPs (CALR and HSPA9) were significantly associated with “Cellular responses to stress” in MS17-treated SW620 cells. CALR is a multifunctional chaperone in the lumen of the endoplasmic reticulum, which can bind with calcium ions and is responsible for protein folding, calcium homeostasis and transcription [161,162,163]. Overexpression of CALR promotes tumor metastasis in CRC cells by regulating calcium ion signaling and cell migration [164,165,166,167]. DNAJA1 belongs to the HSP40 family, which mainly functions as the HSP70 co-chaperone in maintaining the proper folding of other proteins and preventing proteasomal degradation [103,168,169,170]. HSP90AB1 is a molecular chaperone from the HSP90 family that stabilizes proteins in a folded structure and facilitates protein transport and degradation [171]. HSP90AB1 also binds to various proteins, such as steroid hormone receptors, transcription factors, kinases, and ubiquitin ligases, to affect proliferation, metastasis and invasion in cancers [170,172,173,174]. HSPA9 is another compartment-specific member of the HSP70 family, while HSPH1 is a cytoplasmic chaperone acting as a nucleotide exchange factor for HSC70 to prevent protein aggregation and maintain proper folding [103,169,175]. HSPH1 activates the WNT/β-catenin and STAT3 signaling pathways to promote proliferation in CRC [176,177]. MS17 induced upregulation of DNAJA1, HSP90AB1, HSPA1A, HSPA1B and HSPH1, as well as downregulation of CALR and HSPA9 in SW620 cells, which could facilitate the regulation of cellular responses to stress, leading to growth inhibition and apoptosis.

Metabolic enzymes in cluster 5, including the upregulated DEPs (PRDX2 and TKT) and the downregulated TALDO1, were also associated with “Cellular responses to stress” in MS17-treated SW620 cells. PRDX2 is an antioxidant enzyme reported to protect CRC cells from oxidative stress, besides promoting WNT/β-catenin signaling and p53 degradation to enhance cell growth and proliferation [178,179,180]. Interestingly, PRDX2 has dual functions as an oncogene and tumor suppressor in CRC. Overexpression of PRDX2 inhibits TGF-β1-induced EMT and migration in CRC cells [181]. TALDO1 and TKT are glycolytic enzymes in the pentose phosphate pathway, which produce NADPH and pentose sugars for cell proliferation [182]. TALDO1 overexpression has been observed in CRC tissue, while TALDO1 knockdown in lung cancer cells reduced NADPH levels with enhanced ROS accumulation, leading to growth inhibition and apoptosis [183,184]. Overexpression of TKT in CRC cells promotes cell proliferation and metastasis by modulating Akt phosphorylation [185]. TALDO1 and TKT can be regulated by the KEAP1:NFE2L2 pathway in protecting cells against homeostatic responses, such as oxidative, inflammatory, and metabolic stresses [182,186,187,188,189]. MS17 induced the upregulation of PRDX2 and TKT with the TALDO1 downregulation in SW620 cells, which may interfere with the cancer metabolism and increase ROS production to induce cellular stresses, leading to apoptosis.

In the present study, PSMA1 and PSMD13 were the upregulated ubiquitin–protein ligases commonly associated with several pathways in SW620 cells, including “Cellular responses to stress,” “Regulation of expression of SLITs and ROBOs,” and “Metabolism of amino acids and derivatives.” PSMA1 and PSMD13 are proteasome subunits that degrade ubiquitinated proteins in cells, in which proteasomal degradation is essential in almost all cellular processes [146,149,190,191,192,193,194]. Previously, PSMD13 was upregulated in SW480 and SW620 colon cancer cells upon oxaliplatin treatment, leading to growth suppression and apoptosis [195]. SLITs and ROBOs are families of proteins that have been implicated in cancer development and progression by regulating angiogenesis, invasion, metastasis, and immune evasion [196,197,198,199,200,201,202]. PSMA1 and PSMD13 are involved in the regulation of SLITs and ROBOs by modulating protein ubiquitination and proteasomal degradation [203]. Metabolism of amino acids and derivatives, including the catabolism of amino acids, the biosynthesis of the nonessential amino acids and selenocysteine, the urea synthesis and the metabolism of hormones, is essential to maintain a steady supply of amino acids for protein synthesis [204,205,206]. PSMA1 and PSMD13 facilitate ubiquitination and proteasomal degradation to regulate protein levels in every aspect of amino acid metabolism [149]. These findings suggest that MS17 may interfere with ubiquitination and proteasomal degradation by PSMA1 and PSMD13 to target multiple pathways and deregulate the protein expression, which could disrupt cancer progression and eventually lead to apoptosis. In addition, “Metabolism of amino acids and derivatives” in MS17-treated SW620 cells was also significantly associated with another upregulated metabolic enzyme GOT2 in cluster 5. GOT2 catalyzes the inter-conversion and utilization of nitrogenous compounds, which are essential to maintain the balance of amino acids and TCA cycle intermediates [207,208]. It is also a key part of the malate-aspartate shuttle, which is a mechanism that allows cells to transfer reducing equivalents from the cytosol to the mitochondria [209]. GOT2 has been shown to facilitate nitrogen balance in CRC cells by producing amino acids and regulating the urea cycle through an HIF1a–SOX12–GOT2 axis, affecting cell proliferation and metastasis [210]. Our findings suggest that MS17 upregulated GOT2 to affect metabolism of amino acids and derivatives in SW620 cells, which could be associated with growth suppression, anti-proliferation and apoptosis.

Histones H1-2 and H1-3 are two different variants of the linker histone H1 responsible for wrapping DNA around the nucleosome in regulating chromatin dynamics and restricting the DNA accessibility to transcription factors and RNA polymerases. Accumulating evidence has demonstrated the importance of histone H1-2 in regulating gene expression, which affects cellular processes in cancer cells, such as apoptosis, DNA repair, cell proliferation and migration [211,212,213,214,215,216]. Histone H1-3 overexpression in epithelial ovarian cancer cells has been reported to reduce cell proliferation and colony formation by suppressing the noncoding oncogene H19 [217]. Histones H1-2 and H1-3 were downregulated in MS17-treated SW620 cells and significantly associated with “Cellular responses to stress.” Under cellular stress, histone H1 can undergo various modifications, such as acetylation, phosphorylation, methylation, and ubiquitination, which affect its interaction with DNA and other proteins [218,219]. These modifications can alter the chromatin dynamics and the transcriptional stress response [219,220,221,222]. For example, histone H1 acetylation can reduce its affinity for DNA and increase the accessibility of stress-responsive genes [223]. Histone H1 phosphorylation can facilitate the recruitment of DNA repair factors and the activation of cell cycle checkpoints [211,212,214]. These findings suggest that MS17 may downregulate histones H1-2 and H1-3 to disrupt cellular responses to stress, leading to anti-proliferation and apoptosis in SW620 cells.

In MS17-treated SW620 cells, the upregulated DYNC1H1 and VCP in cluster 4 and MSN in cluster 2 were significantly associated with “Cellular responses to stress.” DYNC1H1 is a cytoplasmic dynein involved in intracellular transport of various cargoes along the microtubules in the cell [224]. DYNC1H1 regulates the movement and function of endosomes, which are membrane-bound vesicles that transmit signals from the environment (growth factors, hormones, and nutrients) and modulate various pathways, such as the extracellular signal-regulated kinases (ERKs) 1/2 and the c-Fos transcription factor, that are important for cell survival, growth, differentiation, and adaptation to stress [225,226,227,228,229]. VCP is an ATPase involved in cellular responses to stress by maintaining protein synthesis, folding, and degradation [230]. VCP helps to eliminate misfolded or damaged proteins that accumulate under stress conditions, such as heat shock, oxidative stress and DNA damage [231,232,233]. MSN is a member of the ezrin–radixin–moesin (ERM) family that links the plasma membrane to the actin cytoskeleton [234]. MSN is involved in cellular responses to stress by mediating the changes in cell shape, adhesion, and migration that occur under stress conditions, such as mechanical stress, inflammation, or infection [235,236]. MSN also modulates the signaling pathways that are activated by stress, such as the MAPK, PI3K/Akt, and Rho pathways, by interacting with various receptors, adaptors, and effectors at the membrane–cytoskeleton interface [234,237,238,239]. Therefore, MS17 upregulated DYNC1H1, VCP and MSN in SW620 cells, which may facilitate cellular responses to stress by regulating different aspects of cellular physiology, such as proteostasis, transcription, translation, morphology, adhesion, and migration, which could be associated with anti-cancer effects of MS17.

Moreover, the ribosomal proteins in MS17-treated SW620 cells were also significantly associated with other pathways, including “Eukaryotic translation elongation,” “Nonsense-mediated decay (NMD),” “Regulation of expression of SLITs and ROBOs,” and “Metabolism of amino acids and derivatives.” “Eukaryotic translation elongation” and “Nonsense-mediated decay (NMD)” are two important pathways in gene translation which involve the assembly of ribosomal proteins to form functional subunits. NMD serves as an intracellular mechanism to degrade mRNAs containing premature stop codons, preventing the production of truncated proteins to maintain normal gene expression [240]. Eukaryotic translation elongation requires the ribosome to move along the mRNA (codon recognition) to add amino acids to the growing polypeptide chain during peptide bond formation and translocation [241]. “Eukaryotic translation elongation” was also significantly associated with the upregulated EEF1G in MS17-treated SW620 cells. EEF1G is part of the elongation factor-1 complex that delivers aminoacyl-tRNA and amino acids to the ribosome during the elongation step in gene translation [242]. EEF1G has been reported to be overexpressed in multiple cancers, including CRC, but little is known about its potential roles in cancer progression [243,244]. Additionally, these ribosomal proteins play essential roles in the “Regulation of expression of SLITs and ROBOs” by modulating the translation of ROBO receptors. For instance, the 80S ribosome regulates the translation of ROBO3.2 mRNA containing a premature stop codon, which initiates NMD [245,246,247]. As for the metabolism of amino acids and derivatives, these ribosomal proteins are part of the eukaryotic ribosomes that modulate protein synthesis by facilitating mRNA translation into proteins [138,248,249,250,251,252]. Taken together, MS17 modulated the differential expression of ribosomal proteins and upregulated EEF1G in SW620 cells, which could affect the gene translation, expression of SLITs and ROBOs and metabolism of amino acids and derivatives, resulting in growth inhibition and apoptosis.

“Gene and protein expression by JAK-STAT signaling after interleukin-12 stimulation” were significantly associated with the upregulated HSPA9 and cytoskeletal proteins (LCP1, MSN) and the downregulated TALDO1 in MS17-treated SW620 cells. The JAK-STAT pathway coordinates the intercellular communication between tumor cells and their immune microenvironment, leading to cell proliferation, survival, stemness, immune evasion and tumor progression [253,254]. Interleukin-12 (IL-12) is a cytokine that activates the JAK-STAT signaling and regulates the expression of various immune and inflammatory molecules [255,256]. HSPA9 regulates mitochondrial iron–sulfur cluster (ISC) biogenesis by stabilizing ISC cluster assembly proteins (FXN, NFU1, NFS1 and ISCU1) that are essential for activating many crucial enzymes in JAK-STAT signaling [257,258]. LCP1 is involved in actin polymerization and cytoskeleton remodeling, which also helps to regulate the activation and migration of T cells and macrophages essential for immune responses [259,260]. LCP1 is a downstream target of STAT3 in the JAK-STAT pathway, while MSN is substrate of JAK2 that phosphorylates and activates STAT proteins [261,262]. MSN modulates the signaling and endocytosis of various receptors, including cytokine receptors that activate the JAK-STAT pathway [263]. TALDO1 is a target of STAT5 in the JAK-STAT pathway, which regulates the expression of genes involved in cell cycle, apoptosis, and inflammation [264,265,266]. Our findings suggest that MS17 upregulated HSPPA9, LCP1 and MSN and downregulated TALDO1 in SW620 cells, which could affect the JAK-STAT signaling pathway and associated with apoptosis, proliferation, and immune response, resulting in anti-cancer effects.

## 4. Materials and Methods

### 4.1. Preparation of Curcumin Analogue (MS17) and Curcumin

Curcumin analogue MS17 (1,5-bis(2-hydroxyphenyl)-1,4-pentadiene-3-one) is a chemically purified diarylpentanoid synthesized by coupling the appropriate aromatic aldehyde with acetone under base-catalyzed aldol condensation, using a 1:2 ratio of ketone to aldehyde [70]. The characterization of the analogue was based on the analysis of its spectroscopic data and the comparison of this data with the related compounds [70]. Curcumin was purchased from Nacalai Tesque (Product Grade: Nacalai Special Grade. Nacalai Tesque, Inc., Kyoto, Japan). The 50 mM concentration of MS17 and curcumin stock solutions were prepared in DMSO (Molecular Biology Grade, Sigma Aldrich, St. Louis, MO, USA).

### 4.2. Cell Culture and Maintenance

Normal human lung fibroblast (WI-38, ATCC^®^ CCL-75™), primary (SW480, ATCC^®^ CCL-228™) and metastatic (SW620, ATCC^®^ CCL-227™) human colon cancer cell lines were purchased from the American Type Culture Collection (ATCC, Manassas, VA, USA). WI-38 cells were grown in Eagle’s minimum essential medium (EMEM) (Corning Life Sciences, Corning, NY, USA) and stored in a humidified 37 °C incubator with 5% CO_2_. Both colon cancer cell lines were grown in Leibovitz L-15 medium (Corning Life Sciences, Corning, NY, USA) and stored at 37 °C in a humidified incubator without CO_2_. All media were supplemented with 10% fetal bovine serum (FBS, Gibco, Grand Island, NY, USA) and 1% penicillin (100 U/mL)–streptomycin (100 µg/mL) (Gibco). Cells were regularly monitored to ensure a normal and consistent morphology without contaminants. Cells were maintained by proper aseptic techniques and left to grow until they reached a confluency between 70 to 80%.

### 4.3. Cytotoxicity and Anti-Proliferative Assays of MS17 and Curcumin

Cells were plated in a 96-well culture plate (Nunc, Roskilde, Denmark) in triplicate at a density of 7 × 10^4^ cells/mL. The plate was incubated in a humidified incubator overnight at 37 °C to allow cell attachments to the bottom of the wells. After 24 h, the media was replaced with fresh media containing MS17 or curcumin ranging from 1.56–100 μm and incubated at 37 °C in a humidified incubator. The cells were incubated for 72 h for cytotoxicity analysis and 24, 48 and 72 h for anti-proliferative assay. The MS17 compound was prepared in a stock solution of 50 mM in DMSO (Sigma-Aldrich, St. Louis, MO, USA) and diluted in the culture media to a working concentration of 200 μM. Curcumin was used as a positive control and prepared similarly. A twofold serial dilution was performed in triplicate down the columns of the plate, yielding a final volume and concentration of 200 μL of media with 0.2% DMSO in each well. Control cells were treated with media containing 0.2% of DMSO. The cell viability and anti-proliferative assay were determined by using a 3-(4,5-dimethylthiazol-2-yl)-2,5-diphenyltetrazolium bromide (MTT) assay (Mosmann, 1983). Following incubation, the supernatant was aspirated, and 0.5 mg/mL MTT solution was added and incubated for 4 h at 37 °C. The MTT solution was replaced with 100 μL of DMSO to dissolve the formazan crystal. The absorbance was measured by a BioTek EON (BioTek Instrument, Winooski, VT, USA) microplate reader at a wavelength of 570/650 nm. The equation for cell viability percentage is as follows:

$$Cell\;viability\;(\%)=\frac{Absorbance\;of\;Treated\;Cells}{Absorbance\;of\;Untreated\;Cells}\times 100$$

The half-maximal effective concentration (EC_50_) value was generated using GraphPad Prism version 9.3 (GraphPad Software, San Diego, CA, USA). EC_50_ value represents the concentration at which the tested compound caused a 50% growth inhibitory effect averaged from triplicate absorbance values. Selectivity index (SX) is the degree of selectivity of the compound tested against the cancerous cell, in which SX value above 100 indicates the tested compound possesses a high cytotoxic selectivity compared to normal cells. SX value was determined based on the EC_50_ values obtained from cytotoxicity assays of MS17 against colon cancer cells and normal lung fibroblast cells. SX values were calculated based on the following equation, which was adapted from Popiolkiewicz et al., 2005 [267]:

$$Selective\;Index\;\left(SX\right)=\frac{{EC}_{50}\;(Normal\;cell\;line)}{{EC}_{50}\;(Cancer\;cell\;line)}$$

### 4.4. Induction of Apoptosis with MS17

The apoptotic activity of MS17 against SW480 and SW620 was assessed using morphological evaluation of apoptotic cells and protein capillary electrophoresis to validate the expression of caspase-3, cleaved caspase-3 and Bcl-2. Cancer cells were exposed to MS17 at two different concentrations of 1× EC_50_ [SW480, 4.10 μM; SW620, 2.50 μM] and 2× EC_50_ [SW480, 8.20 μM; SW620, 5 μM] for 24 and 48 h. Each experiment included a set of untreated control cells.

### 4.5. Morphological Observation and Quantitative Analysis of Apoptotic Cells by Acridine Orange–Propidium Iodide Double-Staining Technique Using Fluorescence Microscope

The morphological assessment of cell death was performed using acridine orange (AO) and propidium iodide (PI) double staining. This method was used to differentiate between viable and non-viable cells and cells that undergo early or late apoptosis and necrosis. Cells were seeded in T-25 flasks (SPL, Pochon, Kyonggi-do, South Korea) and incubated 24 h before treatment. Cells were treated with two concentrations of MS17 [1× EC_50_ and 2× EC_50_] at 24, 48 and 72 h. Upon the end of incubation, the cells were washed with PBS and resuspended in 100 μL of ice-cold PBS. A mixture of the fluorescent dye staining solution (1:1), comprising 50 μg/mL of acridine orange (AO) and 50 μg/mL of propidium iodide (PI), was freshly prepared. AO-PI mixture (10 µL) was added to the cell suspension and incubated at room temperature in the dark for 5 min. The freshly stained cell suspension was dropped onto a glass slide and observed under a UV-fluorescence microscope (Olympus BX41) attached with Leica LAS X software version 3.7.6. Fluorescent images were captured using a dual-filter set for FITC (green) and rhodamine (red). Upon completion, a minimum of 200 cells were counted per sample and the percentage of each cell type (viable, apoptotic, and necrotic cells) from each population was calculated based on the following equation:

$$Percentage\;of\;cells\;(\%)=\frac{Number\;of\;cells\;(viable/apoptotic/necrotic)}{Total\;target\;cells}\times 100$$

The different morphological criteria were used to clarify healthy live cells, early or late apoptotic and necrotic cells based on reference protocols as previously described [63,268].

### 4.6. Measurement of Caspase-3 Activity in MS17-Treated SW480 and SW620 Colon Cancer Cells

Caspase-3 activity in MS17-treated SW480 and SW620 cells was evaluated using the Caspase-3 Colorimetric Assay Kit (Raybiotech Inc., Peachtree Corners, GA, USA) based on the manufacturer protocol. Firstly, the cancer cells were seeded in T75 flasks (Nunc) and grown for 24 h. The cells were exposed to MS17 treatment (1× and 2× EC_50_ concentrations) for 24, 48 and 72 h. Following treatment, cells were washed with PBS and resuspended in 50 µL of ice-cold lysis buffer. The cell lysate and lysis buffer mixture were incubated for 10 min on ice. After incubation, the cells were spun at 10,000× *g* for 1 min. Protein concentration was measured using the Pierce BCA Protein Assay Kit (Thermo Fisher Scientific, Waltham, MA, USA). Next, 200 µg of protein lysate was diluted to 50 µL of cell lysis buffer for each replicate. Then, 50 µL of 2× reaction buffer followed by 5 µL of 4 mM DEVD-pNA substrate were added into each assay. The assay was incubated at 37 °C for 2 h. The intensity of the color development was measured at 405 nm using a microplate spectrophotometer (BioTek™ EON™ Microplate Spectrophotometers, Fisher Scientific, Waltham, MA, USA). Caspase-3 activity was presented in the fold change of absorbance from treated cells against absorbance from untreated cells (control):

Fold Change = Absorbance of Treated Cells/Absorbance of Untreated Cells

### 4.7. Quantification of Bcl-2 Protein Concentration in MS17-Treated SW480 and SW620 Colon Cancer Cells

Bcl-2 cellular protein concentration was quantified using the Bcl-2 ELISA kit (Invitrogen, Thermo Fisher Scientific, Waltham, MA, USA) following the manufacturer’s protocol. Each well of the supplied microtiter was pre-coated with anti-human Bcl-2 antibody. The cells were seeded in T75 flasks (Nunc) and incubated for 24 h before treated with two MS17 concentrations (1× and 2× EC_50_) for 24 and 48 h. Upon the end of treatment, the cells were washed with PBS and resuspended in lysis buffer. The protein concentration was determined using the Pierce BCA Protein Assay Kit (Thermo Scientific, Waltham, MA, USA). Firstly, standards and samples were added to the wells, followed by the loading of biotin conjugate. The plate was incubated under room temperature on a microplate shaker for 2 h. After that, the wells were washed to remove unbound material. Streptavidin–HRP was added into each well and continued with 1 h incubation at room temperature. Then, the wells were washed again to remove unbound material. TMB substrate was added into each well to react with the HRP enzyme, resulting in color development. Finally, stop solution was added to terminate the color development, and the color intensity was measured at 450 nm using a microplate spectrophotometer (BioTek™ EON™ Microplate Spectrophotometers, Fisher Scientific, Waltham, MA, USA). Data was presented in the fold change of absorbance from treated cells against absorbance from untreated cells (control).

### 4.8. MS17 Treatment for Proteomic Analysis

Both SW480 and SW620 cells were seeded at a density of 1 × 10^6^ cells in T-75 flasks and incubated overnight to allow cell attachment at 37 °C in a humidified incubator. After that, the cells were incubated with fresh media containing MS17 of 2× EC_50_ concentrations (SW480, 8.20 μM; SW620, 5 μM) for 24 h. Meanwhile, the untreated cells received fresh media with 0.2% DMSO. Upon the end of treatment, the cells were harvested by pre-warmed Accutase^®^ Cell Detachment Solution (Innovative Cell Technologies, San Diego, CA, USA), washed with 2 mL of 1× PBS (Cornihng^®^, Corning, NY, USA) and centrifuged twice to obtain a fresh, clean pellet. The fresh pellet was subjected to total protein extraction using ice-cold RIPA buffer (Nacalai Tesque Inc., Kyoto, Japan) supplemented with EDTA-free Halt™ protease and phosphatase inhibitor cocktail (Sigma-Aldrich, St. Louis, MO, USA). The protein concentration was quantified using the Pierce BCA Protein Assay Kit (Thermo Scientific, Waltham, MA, USA). The experiment was performed in three biological replicates.

### 4.9. In-Solution Digestion and Cleanup

For each sample, 100 μg of protein lysate was subjected to in-solution digestion. First, 25 μL of 100 mM ammonium bicarbonate (ABC) (MD Millipore, Darmstadt, Germany), 25 μL of tetrafluoroethylene (TFE) (Millipore, Darmstadt, Germany), and 1 μL of 200 mM dithiothreitol (DTT) (EMD Millipore, Darmstadt, Germany) were added to each protein sample. The sample mixture was vortexed and heated at 90 °C for an hour. The sample was left to cool to room temperature, followed by adding 4 μL of 200 mM iodoacetamide (IAM) (GE Healthcare, Buckinghamshire, UK) to the mixture. The sample was incubated in the dark at room temperature for 1 h. After that, 1 μL of DTT was added to the sample mixture, vortexed to mix well, and incubated in the dark at room temperature for 1 h to quench excess IAM in the sample mixture. The sample mixture was diluted by adding 300 μL of MilliQ water and 100 μL of ABC. Then, 1 μL of MS Grade Pierce Trypsin Protease (Thermo Fisher Scientific, Waltham, MA, USA) was added to the mixture, and the samples were incubated at 37 °C for 18 h. Later, 1 μL of formic acid was added to stop the trypsin reaction. The sample was dried using centrifugal evaporator CVE-3100 (Eyela, Tokyo, Japan) and desalted through a C18 spin column (Pierce Biotechnology, Thermo Fisher Scientific, Rockford, IL, USA) according to the manufacturer’s protocol.

### 4.10. Liquid Chromatography–Tandem Mass Spectrometry (LCMS/MS) Analysis

The dried, desalted peptide samples were reconstituted with 20 μL of 0.1% formic acid prior to being analyzed on nano-ESI-QTOF (Agilent, 65000 iFunnel Q-TOF LC/MS) equipped with Agilent Large Capacity Chip (G4240-62010 300Å-C18) that was equilibrated with 0.1% formic acid (solution A). 10 μL of peptide sample was injected and eluted from the column at a flow rate of 500 nL/min with an increase in 5 to 70% of solution B (acetonitrile in water with 0.1% formic acid) for 60 min. The QTOF polarity was positive, while the capillary and fragmenter voltages were 1900 V and 360 V, respectively. The drying gas flowed through the column at 325 °C with a flow rate of 5.0 L/min. The spectra were acquired in auto MS/MS mode in a mass range of 110–3000 (*m*/*z*) for the MS scan and 50–3000 (*m*/*z*) for the MS/MS scan. The spectra were then analyzed using PEAKS X+ software (Bioinformatics Solutions Inc., Waterloo, ON, Canada).

### 4.11. Total Protein Identification and Label-Free Quantification

Protein identification in both control and treated samples was performed via automated de novo sequencing using PEAKS X+ software. The peptide spectra were matched against in silico-digested *Homo sapiens* database downloaded from UniProt (https://www.uniprot.org/) [release 2023_04, accessed on 26 April 2023]. A mass shift of 57.02 Da (carbamidomethylation), fragment mass tolerance of 0.1 Da, monoisotopic precursor, and false-discovery rate (FDR) less than 1.0% were set as the search criterion and trypsin as the digestion enzyme. The mass error tolerance was 20 ppm, and the retention time shift tolerance was 6 min. At FDR less than 1.0%, proteins that passed through significance (−10logP) = 13 or equivalent to a p-value of less than 0.05, with at least one unique peptide, and a ratio less than 0.90 or more than 1.10 were selected as query terms for subsequent bioinformatics analysis. Label-free quantification (LFQ) was performed on the PEAKS Q module embedded in PEAKS X+ software version 10.5 to determine the protein expression profile of each sample. The ratio is defined as the protein intensity of the treated sample vs. the control sample.

### 4.12. Bioinformatic Analysis

All DEPs obtained from the LFQ analysis were classified into their respective PANTHER protein classes (http://www.pantherdb.org/) [version 17.0, accessed on 11 July 2023] Subsequently, statistical enrichment analysis for the biological process, molecular function, and cellular component of the DEP against the gene ontology (GO) consortium database (http://geneontology.org/) [version 2023-06-11, accessed on 20 July 2023] was performed using the BiNGO plugin (3.0.3) in Cytoscape (3.9.1) environment. The enriched GO terms were filtered from FDR ≤ 0.05 as the statistically significant cutoff upon the right-sided hypergeometric multiple correction testing with the Benjamini–Hochberg option. The protein–protein interaction (PPI) network of all DEPs was constructed from STRING (Search Tool for the Retrieval of INteracting Genes/Proteins) [https://string-db.org/] [version 12, accessed on 28 July 2023], which maps the DEPs against the *Homo sapiens* database at high confidence score (0.70). To further identify highly connected protein clusters within the network, k-means clustering analysis was performed. The DEPs identified were mapped to the Reactome pathways (https://reactome.org/) [version 85.0, accessed on 27 July 2023], in which an overrepresentation test at FDR < 0.05 as the criterion was performed to select the associated enriched Reactome pathways.

### 4.13. Statistical Analysis

All experiments were conducted in triplicate. Data are presented as means ± standard error of the mean (SEM) from three biological replicates. Comparison between the datasets was performed using one-way and two-way analysis of variance (ANOVA) followed by Dunnett’s multiple-group comparison test. Statistically significant differences between groups were set at *p* ≤ 0.05. All statistical analysis was performed using GraphPad Prism Version 9.3 (GraphPad Software Inc., La Jolla, CA, USA).

## 5. Conclusions

MS17 as a synthetic analogue is comparable to the parent compound, curcumin to demonstrate a higher cytotoxicity and anti-proliferative effect in SW480 and SW620 colon cancer cells. Our morphological analysis indicated that MS17 induced apoptotic changes in both cell lines in time- and dose-dependent effects while there was no significance in caspase-3 activity or downregulation of Bcl-2 protein. Further analysis of the DEPs regulated by MS17 suggests that MS17 has a higher potency than curcumin to target multiple molecular pathways in SW480 and SW620 cells. Based on proteomic profiling, we identified several DEPs associated with the top enriched molecular pathways that could play an important role in inducing apoptosis and anti-cancer effects in SW480 and SW620 cells upon MS17 treatment. Selected DEPs, such as RPL and RPS ribosomal proteins, heat shock proteins (HSPs) and ubiquitin–protein ligases (UBB and UBC) which were significantly associated with “Cellular responses to stress” may facilitate the cytotoxicity, anti-proliferative and apoptotic activities of MS17 in SW480 and SW620 cells. Further investigation is essential to determine the alternative apoptotic mechanisms of MS17 that are independent of caspase-3 activity and Bcl-2 protein expression in SW480 and SW620 cells. MS17 could be a potential anti-cancer agent in primary and metastatic colon cancer cells.

## Figures and Tables

**Table 1:** Enriched Reactome pathways mapped by all DEPs identified in MS17-treated SW480 cells upon 8.20 µM of MS17 treatment for 24 h.

Pathway Identifier	Pathway Name	Entities FDR	Submitted Entities Found	Protein Class	Cluster
R-HSA-2262752	Cellular responses to stress	1.23 × 10^−14^	HSP90AA1, HSPA5, HSPA1B, HSPA1A	Chaperone	Cluster 1
			RPL7A, RPS27A, UBA52	Ribosomal protein	Cluster 2
			UBB, UBC	Ubiquitin–protein ligase	
R-HSA-8948747	Regulation of PTEN localization	1.35 × 10^−6^	RPS27A, UBA52	Ribosomal protein	Cluster 2
			UBB, UBC	Ubiquitin–protein ligase	
R-HSA-1253288	Downregulation of ERBB4 signaling	1.55 × 10^−6^	RPS27A, UBA52	Ribosomal protein	Cluster 2
R-HSA-1643713	Signaling by EGFR in Cancer	1.60 × 10^−6^	HSP90AA1	Chaperone	Cluster 1
			RPS27A, UBA52	Ribosomal protein	Cluster 2
			UBB, UBC	Ubiquitin–protein ligase	
R-HSA-8863795	Downregulation of ERBB2 signaling	2.06 × 10^−6^	HSP90AA1	Chaperone	Cluster 1
			RPS27A, UBA52	Ribosomal protein	Cluster 2
			UBB, UBC	Ubiquitin–protein ligase	
R-HSA-2691230	Signaling by NOTCH1 HD Domain mutants in Cancer	2.41 × 10^−6^	RPS27A, UBA52	Ribosomal protein	Cluster 2
			UBB, UBC	Ubiquitin–protein ligase	

**Table 2:** Enriched Reactome pathways mapped by all DEPs identified in MS17-treated SW620 cells upon 5 µM of MS17 treatment for 24 h.

Pathway Identifier	Pathway Name	Entities FDR	Submitted Entities Found	Protein Class	Cluster
R-HSA-2262752	Cellular responses to stress	2.78 × 10^−15^	RPS9, RPS6, RPS27L, RPS3A, RPL10A, RPL8, RPL6, RPS15, RPL7A, RPS28, RPS27, RPLP2, RPS20, RPL26, RPS11, RPS10, RPL26L1, RPS12	Ribosomal protein	Cluster 1
			CALR, DNAJA1, HSP90AB1, HSPA1A, HSPA1B, HSPA9, HSPH1	Chaperone	Cluster 2
			VCP	Transport protein	
			PSMA1, PSMD13,	Ubiquitin–protein ligase	
			H1-3, H1-2,	Chromatin-regulatory	Cluster 3
			MSN	Cytoskeletal protein	Cluster 4
			DYNC1H1	DNA binding protein	
			PRDX2, TALDO1, TKT	Metabolic enzyme	Cluster 5
R-HSA-156842	Eukaryotic Translation Elongation	2.78 × 10^−15^	RPS9, RPS6, RPS27L, RPS3A, RPL10A, RPL8, RPL6, RPS15, RPL7A, RPS28, RPS27, RPLP2, RPS20, RPL26, RPS11, RPS10, RPL26L1, RPS12	Ribosomal protein	Cluster 1
			EEF1G	RNA-binding protein	Cluster 2
R-HSA-927802	Nonsense-Mediated Decay (NMD)	2.78 × 10^−15^	RPS9, RPS6, RPS27L, RPS3A, RPL10A, RPL8, RPL6, RPS15, RPL7A, RPS28, RPS27, RPLP2, RPS20, RPL26, RPS11, RPS10, RPL26L1, RPS12	Ribosomal protein	Cluster 1
R-HSA-9010553	Regulation of expression of SLITs and ROBOs	2.78 × 10^−15^	RPS9, RPS6, RPS27L, RPS3A, RPL10A, RPL8, RPL6, RPS15, RPL7A, RPS28, RPS27, RPLP2, RPS20, RPL26, RPS11, RPS10, RPL26L1, RPS12	Ribosomal protein	Cluster 1
			PSMA1, PSMD13	Ubiquitin–protein ligase	Cluster 2
R-HSA-71291	Metabolism of amino acids and derivatives	4.78 × 10^−8^	RPS9, RPS6, RPS27L, RPS3A, RPL10A, RPL8, RPL6, RPS15, RPL7A, RPS28, RPS27, RPLP2, RPS20, RPL26, RPS11, RPS10, RPL26L1, RPS12	Ribosomal protein	Cluster 1
			GOT2	Metabolic enzyme	Cluster 5
			PSMA1, PSMD13	Ubiquitin–protein ligase	Cluster 2
R-HSA-8950505	Gene and protein expression by JAK-STAT signaling after Interleukin-12 stimulation	2.27 × 10^−6^	HSPA9	Chaperone	Cluster 2
			LCP1, MSN	Cytoskeletal protein	Cluster 4
			TALDO1	Metabolic enzyme	Cluster 5

## Data Availability

The data presented in this study are available within the article.

## Supplementary Materials

The following supporting information can be downloaded at https://www.mdpi.com/article/10.3390/ijms25063503/s1.

## References

[1] Sung H., Ferlay J., Siegel R.L., Laversanne M., Soerjomataram I., Jemal A., Bray F., Bsc M.F.B., Me J.F., Soerjomataram M.I. (2021). Global Cancer Statistics 2020: GLOBOCAN Estimates of Incidence and Mortality Worldwide for 36 Cancers in 185 Countries. CA Cancer J. Clin..

[2] Arnold M., Sierra M.S., Laversanne M., Soerjomataram I., Jemal A., Bray F. (2017). Global patterns and trends in colorectal cancer incidence and mortality. Gut.

[3] Hassan M.R.A., Ismail I., Suan M.A.M., Ahmad F., Khazim W.K.W., Othman Z., Said R.M., Tan W.L., Rahmah S., Mohammed N.S. (2016). Incidence and mortality rates of colorectal cancer in Malaysia. Epidemiol. Health.

[4] Ramzi N.H., Chahil J.K., Lye S.H., Munretnam K., Sahadevappa K.I., Velapasamy S., Hashim N.A.N., Cheah S.K., Lim G.C.C., Hussein H. (2014). Role of genetic & environment risk factors in the aetiology of colorectal cancer in Malaysia. Indian J. Med. Res..

[5] Veettil S.K., Lim K.G., Chaiyakunapruk N., Ching S.M., Abu Hassan M.R. (2017). Colorectal cancer in Malaysia: Its burden and implications for a multiethnic country. Asian J. Surg..

[6] Wolpin B.M., Meyerhardt J.A., Mamon H.J., Mayer R.J. (2007). Adjuvant Treatment of Colorectal Cancer. CA Cancer J. Clin..

[7] McQuade M.R., Stojanovska V., Bornstein C.J., Nurgali K. (2017). Colorectal Cancer Chemotherapy: The Evolution of Treatment and New Approaches. Curr. Med. Chem..

[8] Vodenkova S., Buchler T., Cervena K., Veskrnova V., Vodicka P., Vymetalkova V. (2020). 5-fluorouracil and other fluoropyrimidines in colorectal cancer: Past, present and future. Pharmacol. Ther..

[9] Wang M.-T., Jiang H., Boral D., Nie D., Bonavida B. (2013). Cancer Stem Cells in Resistance to Cytotoxic Drugs: Implications in Chemotherapy. Molecular Mechanisms of Tumor Cell Resistance to Chemotherapy: Targeted Therapies to Reverse Resistance.

[10] To K.K.W., Wu M., Tong C.W.S., Yan W., Cho C.H., Hu T. (2020). Drug transporters in the development of multidrug resistance in colorectal cancer. Drug Resistance in Colorectal Cancer: Molecular Mechanisms and Therapeutic Strategies.

[11] Phipps A.I., Limburg P.J., Baron J.A., Burnett-Hartman A.N., Weisenberger D.J., Laird P.W., Sinicrope F.A., Rosty C., Buchanan D.D., Potter J.D. (2015). Association Between Molecular Subtypes of Colorectal Cancer and Patient Survival. Gastroenterology.

[12] Wu J.B., Li X.J., Liu H., Liu Y.J., Liu X.P. (2023). Association of KRAS, NRAS, BRAF and PIK3CA gene mutations with clinicopathological features, prognosis and ring finger protein 215 expression in patients with colorectal cancer. Biomed. Rep..

[13] Włodarczyk M., Włodarczyk J., Siwiński P., Sobolewska-Włodarczyk A., Fichna J. (2018). Genetic Molecular Subtypes in Optimizing Personalized Therapy for Metastatic Colorectal Cancer. Curr. Drug Targets.

[14] Feng F., Sun H., Zhao Z., Sun C., Zhao Y., Lin H., Yang J., Xiao Y., Wang W., Wu D. (2022). Identification of APC Mutation as a Potential Predictor for Immunotherapy in Colorectal Cancer. J. Oncol..

[15] Lestari M.L.A.D., Indrayanto G., Brittain H.G. (2014). Chapter Three—Curcumin. Profiles of Drug Substances, Excipients and Related Methodology.

[16] Mosieniak G., Adamowicz M., Alster O., Jaskowiak H., Szczepankiewicz A.A., Wilczynski G.M., Ciechomska I.A., Sikora E. (2012). Curcumin induces permanent growth arrest of human colon cancer cells: Link between senescence and autophagy. Mech. Ageing Dev..

[17] Lim T.G., Lee S.Y., Huang Z., Lim D.Y., Chen H., Jung S.K., Bode A.M., Lee K.W., Dong Z. (2014). Curcumin Suppresses Proliferation of Colon Cancer Cells by Targeting CDK2Curcumin Inhibits CDK2 to Suppress Colon Cancer Cell Growth. Cancer Prev. Res..

[18] Ismail N.I., Othman I., Abas F., Lajis N.H., Naidu R. (2019). Mechanism of Apoptosis Induced by Curcumin in Colorectal Cancer. Int. J. Mol. Sci..

[19] Reyhaneh M.-M., Seyed Mahdi H., Soodabeh S., Amir A., Majid K. (2018). Curcumin Effects on the Wnt Signaling Pathway in Colorectal Cancer Stem Cells. Basic Clin. Cancer Res..

[20] Weng W., Goel A. (2022). Curcumin and colorectal cancer: An update and current perspective on this natural medicine. Semin. Cancer Biol..

[21] Villota H., Röthlisberger S., Pedroza-Díaz J. (2022). Modulation of the Canonical Wnt Signaling Pathway by Dietary Polyphenols, an Opportunity for Colorectal Cancer Chemoprevention and Treatment. Nutr. Cancer.

[22] Toden S., Okugawa Y., Buhrmann C., Nattamai D., Anguiano E., Baldwin N., Shakibaei M., Boland C.R., Goel A. (2015). Novel evidence for curcumin and boswellic acid–induced chemoprevention through regulation of miR-34a and miR-27a in colorectal cancer. Cancer Prev. Res..

[23] Mudduluru G., George-William J.N., Muppala S., Asangani I.A., Kumarswamy R., Nelson L.D., Allgayer H. (2011). Curcumin regulates miR-21 expression and inhibits invasion and metastasis in colorectal cancer. Biosci. Rep..

[24] Gandhy S.U., Kim K., Larsen L., Rosengren R.J., Safe S. (2012). Curcumin and synthetic analogs induce reactive oxygen species and decreases specificity protein (Sp) transcription factors by targeting microRNAs. BMC Cancer.

[25] Shakibaei M., Mobasheri A., Lueders C., Busch F., Shayan P., Goel A. (2013). Curcumin enhances the effect of chemotherapy against colorectal cancer cells by inhibition of NF-κB and Src protein kinase signaling pathways. PLoS ONE.

[26] Howells L.M., Iwuji C.O.O., Irving G.R.B., Barber S., Walter H., Sidat Z., Griffin-Teall N., Singh R., Foreman N., Patel S.R. (2019). Curcumin Combined with FOLFOX Chemotherapy Is Safe and Tolerable in Patients with Metastatic Colorectal Cancer in a Randomized Phase IIa Trial. J. Nutr..

[27] Fan W.-H., Wang F.-C., Jin Z., Zhu L., Zhang J.-X. (2021). Curcumin Synergizes with Cisplatin to Inhibit Colon Cancer through Targeting the MicroRNA-137-Glutaminase Axis. Curr. Med. Sci..

[28] Abdul Satar N., Ismail M.N., Yahaya B.H. (2021). Synergistic Roles of Curcumin in Sensitising the Cisplatin Effect on a Cancer Stem Cell-Like Population Derived from Non-Small Cell Lung Cancer Cell Lines. Molecules.

[29] Liu W., Zhai Y., Heng X., Che F.Y., Chen W., Sun D., Zhai G. (2016). Oral bioavailability of curcumin: Problems and advancements. J. Drug Target..

[30] Kunnumakkara A.B., Harsha C., Banik K., Vikkurthi R., Sailo B.L., Bordoloi D., Gupta S.C., Aggarwal B.B. (2019). Is curcumin bioavailability a problem in humans: Lessons from clinical trials. Expert Opin. Drug Metab. Toxicol..

[31] Mansouri K., Rasoulpoor S., Daneshkhah A., Abolfathi S., Salari N., Mohammadi M., Shabani S. (2020). Clinical effects of curcumin in enhancing cancer therapy: A systematic review. BMC Cancer.

[32] Alven S., Aderibigbe B.A. (2020). Efficacy of Polymer-Based Nanocarriers for Co-Delivery of Curcumin and Selected Anticancer Drugs. Nanomaterials.

[33] Anthwal A., Thakur B.K., Rawat M.S.M., Rawat D.S., Tyagi A.K., Aggarwal B.B. (2014). Synthesis, characterization and in vitro anticancer activity of C-5 curcumin analogues with potential to inhibit TNF-α-induced NF-κB activation. BioMed Res. Int..

[34] Arshad L., Haque M.A., Bukhari S.N.A., Jantan I. (2017). An overview of structure–activity relationship studies of curcumin analogs as antioxidant and anti-inflammatory agents. Future Med. Chem..

[35] Cavaleri F. (2018). Presenting a New Standard Drug Model for Turmeric and Its Prized Extract, Curcumin. Int. J. Inflamm..

[36] Chainoglou E., Hadjipavlou-Litina D. (2019). Curcumin analogues and derivatives with anti-proliferative and anti-inflammatory activity: Structural characteristics and molecular targets. Expert Opin. Drug Discov..

[37] Charan T.R., Bhutto M.A., Bhutto M.A., Tunio A.A., Khuhro G.M., Khaskheli S.A., Mughal A.A. (2021). “Nanomaterials of curcumin-hyaluronic acid”: Their various methods of formulations, clinical and therapeutic applications, present gap, and future directions. Future J. Pharm. Sci..

[38] Ciochina R., Savella C., Cote B., Chang D., Rao D. (2014). Synthesis and Characterization of New Curcumin Derivatives as Potential Chemotherapeutic and Antioxidant Agents. Drug Dev. Res..

[39] D’Angelo N.A., Noronha M.A., Kurnik I.S., Câmara M.C., Vieira J.M., Abrunhosa L., Martins J.T., Alves T.F., Tundisi L.L., Ataide J.A. (2021). Curcumin encapsulation in nanostructures for cancer therapy: A 10-year overview. Int. J. Pharm..

[40] Kabir T., Rahman H., Akter R., Behl T., Kaushik D., Mittal V., Pandey P., Akhtar M.F., Saleem A., Albadrani G.M. (2021). Potential Role of Curcumin and Its Nanoformulations to Treat Various Types of Cancers. Biomolecules.

[41] Karthikeyan A., Senthil N., Min T. (2020). Nanocurcumin: A Promising Candidate for Therapeutic Applications. Front. Pharmacol..

[42] Gupta A.P., Khan S., Manzoor M.M., Yadav A.K., Sharma G., Anand R., Gupta S., Atta ur R. (2017). Chapter 10—Anticancer Curcumin: Natural Analogues and Structure-Activity Relationship. Studies in Natural Products Chemistry.

[43] Shahriari M., Kesharwani P., Johnston T.P., Sahebkar A. (2023). Anticancer potential of curcumin-cyclodextrin complexes and their pharmacokinetic properties. Int. J. Pharm..

[44] Nag A., Chakraborty P., Natarajan G., Baksi A., Mudedla S.K., Subramanian V., Pradeep T. (2018). Bent Keto Form of Curcumin, Preferential Stabilization of Enol by Piperine, and Isomers of Curcumin∩Cyclodextrin Complexes: Insights from Ion Mobility Mass Spectrometry. Anal. Chem..

[45] Alizadeh N., Malakzadeh S. (2020). Changes in chemical stability and bioactivities of curcumin by forming inclusion complexes of beta- and Gama-cyclodextrins. J. Polym. Res..

[46] Khudhayer Oglah M., Fakri Mustafa Y. (2020). Curcumin analogs: Synthesis and biological activities. Med. Chem. Res..

[47] Tabanelli R., Brogi S., Calderone V. (2021). Improving Curcumin Bioavailability: Current strategies and future perspectives. Pharmaceutics.

[48] Joshi P., Verma K., Kumar Semwal D., Dwivedi J., Sharma S. (2023). Mechanism insights of curcumin and its analogues in cancer: An update. Phytother. Res..

[49] Clariano M., Marques V., Vaz J., Awam S., Afonso M.B., Perry M.J., Rodrigues C.M.P. (2023). Monocarbonyl Analogs of Curcumin with Potential to Treat Colorectal Cancer. Chem. Biodivers..

[50] Padhye S., Yang H., Jamadar A., Cui Q.C., Chavan D., Dominiak K., McKinney J., Banerjee S., Dou Q.P., Sarkar F.H. (2009). New difluoro Knoevenagel condensates of curcumin, their Schiff bases and copper complexes as proteasome inhibitors and apoptosis inducers in cancer cells. Pharm. Res..

[51] Huber I., Zupkó I., Gyovai A., Horváth P., Kiss E., Gulyás-Fekete G., Schmidt J., Perjési P. (2019). A novel cluster of C5-curcuminoids: Design, synthesis, in vitro antiproliferative activity and DNA binding of bis(arylidene)-4-cyclanone derivatives based on 4-hydroxycyclohexanone scaffold. Res. Chem. Intermed..

[52] Citalingam K., Abas F., Lajis N.H., Othman I., Naidu R. (2015). Anti-proliferative effect and induction of apoptosis in androgen-independent human prostate cancer cells by 1,5-bis(2-hydroxyphenyl)-1,4-pentadiene-3-one. Molecules.

[53] Nagaraju G.P., Benton L., Bethi S.R., Shoji M., El-Rayes B.F. (2019). Curcumin analogs: Their roles in pancreatic cancer growth and metastasis. Int. J. Cancer.

[54] Noureddin S.A., El-Shishtawy R.M., Al-Footy K.O. (2019). Curcumin analogues and their hybrid molecules as multifunctional drugs. Eur. J. Med. Chem..

[55] Friedman L., Lin L., Ball S., Bekaii-Saab T., Fuchs J., Li P.-K., Li C., Lin J. (2009). Curcumin analogues exhibit enhanced growth suppressive activity in human pancreatic cancer cells. Anti-Cancer Drugs.

[56] Thomas S.L., Zhong D., Zhou W., Malik S., Liotta D., Snyder J.P., Hamel E., Giannakakou P. (2008). EF24, a novel curcumin analog, disrupts the microtubule cytoskeleton and inhibits HIF-1. Cell Cycle.

[57] Adeluola A., Zulfiker A.H.M., Brazeau D., Amin A. (2021). Perspectives for synthetic curcumins in chemoprevention and treatment of cancer: An update with promising analogues. Eur. J. Pharmacol..

[58] Wang Z.S., Chen L.Z., Zhou H.P., Liu X.H., Chen F.H. (2017). Diarylpentadienone derivatives (curcumin analogues): Synthesis and anti-inflammatory activity. Bioorganic Med. Chem. Lett..

[59] Amalraj A., Pius A., Gopi S., Gopi S. (2017). Biological activities of curcuminoids, other biomolecules from turmeric and their derivatives—A review. J. Tradit. Complement. Med..

[60] Novais P., Silva P.M.A., Moreira J., Palmeira A., Amorim I., Pinto M., Cidade H., Bousbaa H. (2021). BP-M345, a New Diarylpentanoid with Promising Antimitotic Activity. Molecules.

[61] Qudjani E., Iman M., Davood A., Ramandi M.F., Shafiee A. (2016). Design and Synthesis of Curcumin-like Diarylpentanoid Analogues as Potential Anticancer Agents. Recent Pat. Anticancer Drug Discov..

[62] Wan Mohd Tajuddin W.N.B., Abas F., Othman I., Naidu R. (2021). Molecular Mechanisms of Antiproliferative and Apoptosis Activity by 1,5-Bis(4-Hydroxy-3-Methoxyphenyl)1,4-Pentadiene-3-one (MS13) on Human Non-Small Cell Lung Cancer Cells. Int. J. Mol. Sci..

[63] Abd Wahab N.A., Abas F., Othman I., Naidu R. (2021). Diarylpentanoid (1,5-bis(4-hydroxy-3-methoxyphenyl)-1,4-pentadiene-3-one) (MS13) Exhibits Anti-proliferative, Apoptosis Induction and Anti-migration Properties on Androgen-independent Human Prostate Cancer by Targeting Cell Cycle-Apoptosis and PI3K Signalling Pathways. Front. Pharmacol..

[64] Ismail N.I., Othman I., Abas F., Lajis N.H., Naidu R. (2020). The Curcumin Analogue, MS13 (1,5-Bis(4-hydroxy-3- methoxyphenyl)-1,4-pentadiene-3-one), Inhibits Cell Proliferation and Induces Apoptosis in Primary and Metastatic Human Colon Cancer Cells. Molecules.

[65] He G., Feng C., Vinothkumar R., Chen W., Dai X., Chen X., Ye Q., Qiu C., Zhou H., Wang Y. (2016). Curcumin analog EF24 induces apoptosis via ROS-dependent mitochondrial dysfunction in human colorectal cancer cells. Cancer Chemother. Pharmacol..

[66] Kanwar S.S., Yu Y., Nautiyal J., Patel B.B., Padhye S., Sarkar F.H., Majumdar A.P.N. (2011). Difluorinated-Curcumin (CDF): A Novel Curcumin Analog is a Potent Inhibitor of Colon Cancer Stem-like Cells. Pharm. Res..

[67] Paulraj F., Abas F., Lajis N.H., Othman I., Hassan S.S., Naidu R. (2015). The Curcumin Analogue 1,5-Bis(2-hydroxyphenyl)-1,4-pentadiene-3-one Induces Apoptosis and Downregulates E6 and E7 Oncogene Expression in HPV16 and HPV18-Infected Cervical Cancer Cells. Molecules.

[68] Lin L., Liu Y., Li H., Li P.-K., Fuchs J., Shibata H., Iwabuchi Y., Lin J. (2011). Targeting colon cancer stem cells using a new curcumin analogue, GO-Y030. Br. J. Cancer.

[69] Citalingam K., Abas F., Lajis N., Othman I., Naidu R. (2018). Identification of commonly regulated protein targets and molecular pathways in PC-3 and DU145 androgen-independent human prostate cancer cells treated with the curcumin analogue 1,5-bis(2-hydroxyphenyl)-1,4-pentadiene-3-one. Asian Pac. J. Trop. Biomed..

[70] Lee K.-H., Aziz F.H.A., Syahida A., Abas F., Shaari K., Israf D.A., Lajis N.H. (2009). Synthesis and biological evaluation of curcumin-like diarylpentanoid analogues for anti-inflammatory, antioxidant and anti-tyrosinase activities. Eur. J. Med. Chem..

[71] Cen L., Hutzen B., Ball S., DeAngelis S., Chen C.-L., Fuchs J.R., Li C., Li P.-K., Lin J. (2009). New structural analogues of curcumin exhibit potent growth suppressive activity in human colorectal carcinoma cells. BMC Cancer.

[72] Jitoe-Masuda A., Fujimoto A., Masuda T. (2013). Curcumin: From Chemistry to Chemistry-Based Functions. Curr. Pharm. Des..

[73] Indira Priyadarsini K. (2013). Chemical and structural features influencing the biological activity of curcumin. Curr. Pharm. Des..

[74] Melcher R., Steinlein C., Feichtinger W., Müller C., Menzel T., Lührs H., Scheppach W., Schmid M. (2000). Spectral karyotyping of the human colon cancer cell lines SW480 and SW620. Cytogenet. Cell Genet..

[75] Hewitt R.E., McMarlin A., Kleiner D., Wersto R., Martin P., Tsoskas M., Stamp G.W., Stetler-Stevenson W.G. (2000). Validation of a model of colon cancer progression. J. Pathol..

[76] Yan W., Yang W., Liu Z., Wu G. (2018). Characterization of microRNA expression in primary human colon adenocarcinoma cells (SW480) and their lymph node metastatic derivatives (SW620). OncoTargets Ther..

[77] Schønberg S.A., Lundemo A.G., Fladvad T., Holmgren K., Bremseth H., Nilsen A., Gederaas O., Tvedt K.E., Egeberg K.W., Krokan H.E. (2006). Closely related colon cancer cell lines display different sensitivity to polyunsaturated fatty acids, accumulate different lipid classes and downregulate sterol regulatory element-binding protein1. FEBS J..

[78] Bauer K.M., Lambert P.A., Hummon A.B. (2012). Comparative label-free LC-MS/MS analysis of colorectal adenocarcinoma and metastatic cells treated with 5-fluorouracil. Proteomics.

[79] Fhaner C.J., Liu S., Ji H., Simpson R.J., Reid G.E. (2012). Comprehensive Lipidome Profiling of Isogenic Primary and Metastatic Colon Adenocarcinoma Cell Lines. Anal. Chem..

[80] McCool E.N., Xu T., Chen W., Beller N.C., Nolan S.M., Hummon A.B., Liu X., Sun L. (2022). Deep top-down proteomics revealed significant proteoform-level differences between metastatic and nonmetastatic colorectal cancer cells. Sci. Adv..

[81] Kubens B.S., Zänker K.S. (1998). Differences in the migration capacity of primary human colon carcinoma cells (SW480) and their lymph node metastatic derivatives (SW620). Cancer Lett..

[82] Siekmann W., Tina E., Koskela von Sydow A., Gupta A. (2019). Effect of lidocaine and ropivacaine on primary (SW480) and metastatic (SW620) colon cancer cell lines. Oncol. Lett..

[83] Abdulrehman G., Xv K., Li Y., Kang L. (2018). Effects of meta-tetrahydroxyphenylchlorin photodynamic therapy on isogenic colorectal cancer SW480 and SW620 cells with different metastatic potentials. Lasers Med. Sci..

[84] Cecilia Subauste M., Kupriyanova T.A., Conn E.M., Ardi V.C., Quigley J.P., Deryugina E.I. (2009). Evaluation of metastatic and angiogenic potentials of human colon carcinoma cells in chick embryo model systems. Clin. Exp. Metastasis.

[85] Rashmi R., Santhosh Kumar T.R., Karunagaran D. (2003). Human colon cancer cells differ in their sensitivity to curcumin-induced apoptosis and heat shock protects them by inhibiting the release of apoptosis-inducing factor and caspases. FEBS Lett..

[86] Sato T., Higuchi Y., Shibagaki Y., Hattori S. (2017). Phosphoproteomic Analysis Identifies Signaling Pathways Regulated by Curcumin in Human Colon Cancer Cells. Anticancer Res..

[87] Hussar P. (2022). Apoptosis Regulators Bcl-2 and Caspase-3. Encyclopedia.

[88] Kirsch D.G., Doseff A., Chau B.N., Lim D.-S., de Souza-Pinto N.C., Hansford R., Kastan M.B., Lazebnik Y.A., Hardwick J.M. (1999). Caspase-3-dependent Cleavage of Bcl-2 Promotes Release of Cytochrome, C. J. Biol. Chem..

[89] Galluzzi L., López-Soto A., Kumar S., Kroemer G. (2016). Caspases Connect Cell-Death Signaling to Organismal Homeostasis. Immunity.

[90] Van Opdenbosch N., Lamkanfi M. (2019). Caspases in Cell Death, Inflammation, and Disease. Immunity.

[91] Nirmala J.G., Lopus M. (2020). Cell death mechanisms in eukaryotes. Cell Biol. Toxicol..

[92] Watson J.L., Hill R., Yaffe P.B., Greenshields A., Walsh M., Lee P.W., Giacomantonio C.A., Hoskin D.W. (2010). Curcumin causes superoxide anion production and p53-independent apoptosis in human colon cancer cells. Cancer Lett..

[93] Song G., Mao Y.B., Cai Q.F., Yao L.M., Ouyang G.L., Bao S.D. (2005). Curcumin induces human HT-29 colon adenocarcinoma cell apoptosis by activating p53 and regulating apoptosis-related protein expression. Braz. J. Med. Biol. Res..

[94] Guo L.-D., Chen X.-J., Hu Y.-H., Yu Z.-J., Wang D., Liu J.-Z. (2013). Curcumin Inhibits Proliferation and Induces Apoptosis of Human Colorectal Cancer Cells by Activating the Mitochondria Apoptotic Pathway. Phytother. Res..

[95] Su C.-C., Lin J.-G., Li T.-M., Chung J.G., Yang J.S., Ip S.-W., Lin W.-C., Chen G.-W. (2006). Curcumin-induced Apoptosis of Human Colon Cancer Colo 205 Cells through the Production of ROS, Ca^2+^ and the Activation of Caspase-3. Anticancer. Res..

[96] Weng Q., Fu L., Chen G., Hui J., Song J., Feng J., Shi D., Cai Y., Ji J., Liang G. (2015). Design, synthesis, and anticancer evaluation of long-chain alkoxylated mono-carbonyl analogues of curcumin. Eur. J. Med. Chem..

[97] Subramaniam D., May R., Sureban S.M., Lee K.B., George R., Kuppusamy P., Ramanujam R.P., Hideg K., Dieckgraefe B.K., Houchen C.W. (2008). Diphenyl Difluoroketone: A Curcumin Derivative with Potent In vivo Anticancer Activity. Cancer Res..

[98] Yang S.-J., Lee S.A., Park M.-G., Kim J.-S., Yu S.-K., Kim C.S., Kim J.-S., Kim S.-G., Oh J.-S., Kim H.-J. (2014). Induction of apoptosis by diphenyldifluoroketone in osteogenic sarcoma cells is associated with activation of caspases. Oncol. Rep..

[99] Lee Y.Q., Rajadurai P., Abas F., Othman I., Naidu R. (2021). Proteomic Analysis on Anti-Proliferative and Apoptosis Effects of Curcumin Analog, 1,5-bis(4-Hydroxy-3-Methyoxyphenyl)-1,4-Pentadiene-3-One-Treated Human Glioblastoma and Neuroblastoma Cells. Front. Mol. Biosci..

[100] Rual J.-F., Venkatesan K., Hao T., Hirozane-Kishikawa T., Dricot A., Li N., Berriz G.F., Gibbons F.D., Dreze M., Ayivi-Guedehoussou N. (2005). Towards a proteome-scale map of the human protein–protein interaction network. Nature.

[101] Raman K. (2010). Construction and analysis of protein–protein interaction networks. Autom. Exp..

[102] Szklarczyk D., Kirsch R., Koutrouli M., Nastou K., Mehryary F., Hachilif R., Gable A.L., Fang T., Doncheva N.T., Pyysalo S. (2022). The STRING database in 2023: Protein–protein association networks and functional enrichment analyses for any sequenced genome of interest. Nucleic Acids Res..

[103] Hu C., Yang J., Qi Z., Wu H., Wang B., Zou F., Mei H., Liu J., Wang W., Liu Q. (2022). Heat shock proteins: Biological functions, pathological roles, and therapeutic opportunities. MedComm.

[104] Arya R., Mallik M., Lakhotia S.C. (2007). Heat shock genes—Integrating cell survival and death. J. Biosci..

[105] Mayer M.P., Bukau B. (2005). Hsp70 chaperones: Cellular functions and molecular mechanism. Cell. Mol. Life Sci..

[106] Wegele H., Müller L., Buchner J. (2004). Hsp70 and Hsp90—A Relay Team for Protein Folding. Reviews of Physiology, Biochemistry and Pharmacology.

[107] Teiten M.-H., Reuter S., Schmucker S., Dicato M., Diederich M. (2009). Induction of heat shock response by curcumin in human leukemia cells. Cancer Lett..

[108] Guo L.D., Shen Y.Q., Zhao X.H., Guo L.J., Yu Z.J., Wang D., Liu L.M., Liu J.Z. (2015). Curcumin Combined with Oxaliplatin Effectively Suppress Colorectal Carcinoma in vivo Through Inducing Apoptosis. Phytother. Res..

[109] Rak S., Čimbora-Zovko T., Gajski G., Dubravčić K., Domijan A.-M., Delaš I., Garaj-Vrhovac V., Batinić D., Sorić J., Osmak M. (2013). Carboplatin resistant human laryngeal carcinoma cells are cross resistant to curcumin due to reduced curcumin accumulation. Toxicol. In Vitro.

[110] Chen H.-W., Yu S.-L., Chen J.J.W., Li H.-N., Lin Y.-C., Yao P.-L., Chou H.-Y., Chien C.-T., Chen W.-J., Lee Y.-T. (2004). Anti-Invasive Gene Expression Profile of Curcumin in Lung Adenocarcinoma Based on a High Throughput Microarray Analysis. Mol. Pharmacol..

[111] Dunsmore K.E., Chen P.G., Wong H.R. (2001). Curcumin, a medicinal herbal compound capable of inducing the heat shock response. Crit. Care Med..

[112] Szebeni G.J., Balázs Á., Madarász I., Pócz G., Ayaydin F., Kanizsai I., Fajka-Boja R., Alföldi R., Hackler L., Puskás L.G. (2017). Achiral Mannich-Base Curcumin Analogs Induce Unfolded Protein Response and Mitochondrial Membrane Depolarization in PANC-1 Cells. Int. J. Mol. Sci..

[113] Roskoski R. (2014). The ErbB/HER family of protein-tyrosine kinases and cancer. Pharmacol. Res..

[114] Wang Z., Wang Z. (2017). ErbB Receptors and Cancer. ErbB Receptor Signaling: Methods and Protocols.

[115] Wang N., Cao Y., Si C., Shao P., Su G., Wang K., Bao J., Yang L. (2022). Emerging Role of ERBB2 in Targeted Therapy for Metastatic Colorectal Cancer: Signaling Pathways to Therapeutic Strategies. Cancers.

[116] Nowak J.A. (2020). HER2 in Colorectal Carcinoma: Are We There yet?. Surg. Pathol. Clin..

[117] Loree J.M., Bailey A.M., Johnson A.M., Yu Y., Wu W., Bristow C.A., Davis J.S., Shaw K.R., Broaddus R., Banks K.C. (2018). Molecular Landscape of ERBB2/ERBB3 Mutated Colorectal Cancer. JNCI J. Natl. Cancer Inst..

[118] Citri A., Gan J., Mosesson Y., Vereb G., Szollosi J., Yarden Y. (2004). Hsp90 restrains ErbB-2/HER2 signalling by limiting heterodimer formation. EMBO Rep..

[119] Segers V.F.M., Dugaucquier L., Feyen E., Shakeri H., De Keulenaer G.W. (2020). The role of ErbB4 in cancer. Cell. Oncol..

[120] Williams C.S., Bernard J.K., Beckler M.D., Almohazey D., Washington M.K., Smith J.J., Frey M.R. (2015). ERBB4 is over-expressed in human colon cancer and enhances cellular transformation. Carcinogenesis.

[121] Frey M.R., Edelblum K.L., Mullane M.T., Liang D., Polk D.B. (2009). The ErbB4 Growth Factor Receptor Is Required for Colon Epithelial Cell Survival in the Presence of TNF. Gastroenterology.

[122] Zhou B., Lin W., Long Y., Yang Y., Zhang H., Wu K., Chu Q. (2022). Notch signaling pathway: Architecture, disease, and therapeutics. Signal Transduct. Target. Ther..

[123] Reedijk M., Odorcic S., Zhang H., Chetty R., Tennert C., Dickson B.C., Lockwood G., Gallinger S., Egan S.E. (2008). Activation of Notch signaling in human colon adenocarcinoma. Int. J. Oncol..

[124] Tyagi A., Sharma A.K., Damodaran C. (2020). A Review on Notch Signaling and Colorectal Cancer. Cells.

[125] Rajendran D.T., Subramaniyan B., Ganeshan M., Nagaraju G.P., Bramhachari P.V. (2017). Role of Notch Signaling in Colorectal Cancer. Role of Transcription Factors in Gastrointestinal Malignancies.

[126] Subramaniam D., Ponnurangam S., Ramamoorthy P., Standing D., Battafarano R.J., Anant S., Sharma P. (2012). Curcumin Induces Cell Death in Esophageal Cancer Cells through Modulating Notch Signaling. PLoS ONE.

[127] Sha J., Li J., Wang W., Pan L., Cheng J., Li L., Zhao H., Lin W. (2016). Curcumin induces G0/G1 arrest and apoptosis in hormone independent prostate cancer DU-145 cells by down regulating Notch signaling. Biomed. Pharmacother..

[128] Zhou S., Zhang S., Shen H., Chen W., Xu H., Chen X., Sun D., Zhong S., Zhao J., Tang J. (2017). Curcumin inhibits cancer progression through regulating expression of microRNAs. Tumor Biol..

[129] Li Y., Zhang J., Ma D., Zhang L., Si M., Yin H., Li J. (2012). Curcumin inhibits proliferation and invasion of osteosarcoma cells through inactivation of Notch-1 signaling. FEBS J..

[130] Liu Z.-C., Yang Z.-X., Zhou J.-S., Zhang H.-T., Huang Q.-K., Dang L.-L., Liu G.-X., Tao K.-S. (2014). Curcumin regulates hepatoma cell proliferation and apoptosis through the Notch signaling pathway. Int. J. Clin. Exp. Med..

[131] Leslie N.R., Kriplani N., Hermida M.A., Alvarez-Garcia V., Wise H.M. (2016). The PTEN protein: Cellular localization and post-translational regulation. Biochem. Soc. Trans..

[132] Milella M., Falcone I., Conciatori F., Cesta Incani U., Del Curatolo A., Inzerilli N., Nuzzo C.M., Vaccaro V., Vari S., Cognetti F. (2015). PTEN: Multiple Functions in Human Malignant Tumors. Front. Oncol..

[133] Serebriiskii I.G., Pavlov V., Tricarico R., Andrianov G., Nicolas E., Parker M.I., Newberg J., Frampton G., Meyer J.E., Golemis E.A. (2022). Comprehensive characterization of PTEN mutational profile in a series of 34,129 colorectal cancers. Nat. Commun..

[134] Abbas Momtazi A., Sahebkar A. (2016). Difluorinated Curcumin: A Promising Curcumin Analogue with Improved Anti-Tumor Activity and Pharmacokinetic Profile. Curr. Pharm. Des..

[135] Roy S., Yu Y., Padhye S.B., Sarkar F.H., Majumdar A.P.N. (2013). Difluorinated-Curcumin (CDF) Restores PTEN Expression in Colon Cancer Cells by Down-Regulating miR-21. PLoS ONE.

[136] Dandawate P.R., Vyas A., Ahmad A., Banerjee S., Deshpande J., Swamy K.V., Jamadar A., Dumhe-Klaire A.C., Padhye S., Sarkar F.H. (2012). Inclusion Complex of Novel Curcumin Analogue CDF and β-Cyclodextrin (1:2) and Its Enhanced In Vivo Anticancer Activity Against Pancreatic Cancer. Pharm. Res..

[137] Saini A.K., Kumar V., Kumar V. (2021). Chapter 2—Ribosome structure. Emerging Concepts in Ribosome Structure, Biogenesis, and Function.

[138] Kumar J., Kumar V., Kumar V. (2021). Chapter 4—Ribosome proteins—Their balanced production. Emerging Concepts in Ribosome Structure, Biogenesis, and Function.

[139] Baker R.T., Board P.G. (1991). The human ubiquitin-52 amino acid fusion protein gene shares several structural features with mammalian ribosomal protein genes. Nucleic Acids Res..

[140] Barnard G.F., Mori M., Staniunas R.J., Begum N.A., Bao S., Puder M., Cobb J., Redman K.L., Steele G.D., Chen L.B. (1995). Ubiquitin fusion proteins are overexpressed in colon cancer but not in gastric cancer. Biochim. Biophys. Acta (BBA)-Mol. Basis Dis..

[141] Luo J., Zhao H., Chen L., Liu M. (2023). Multifaceted functions of RPS27a: An unconventional ribosomal protein. J. Cell. Physiol..

[142] Scarpa E.S., Tasini F., Crinelli R., Ceccarini C., Magnani M., Bianchi M. (2020). The Ubiquitin Gene Expression Pattern and Sensitivity to UBB and UBC Knockdown Differentiate Primary 23132/87 and Metastatic MKN45 Gastric Cancer Cells. Int. J. Mol. Sci..

[143] Chen Z.J., Sun L.J. (2009). Nonproteolytic Functions of Ubiquitin in Cell Signaling. Mol. Cell.

[144] Demasi M., da Cunha F.M. (2018). The physiological role of the free 20S proteasome in protein degradation: A critical review. Biochim. Biophys. Acta (BBA)-Gen. Subj..

[145] Sahu I., Glickman M.H. (2021). Proteasome in action: Substrate degradation by the 26S proteasome. Biochem. Soc. Trans..

[146] Budenholzer L., Cheng C.L., Li Y., Hochstrasser M. (2017). Proteasome Structure and Assembly. J. Mol. Biol..

[147] Sun T., Liu Z., Yang Q. (2020). The role of ubiquitination and deubiquitination in cancer metabolism. Mol. Cancer.

[148] Deng L., Meng T., Chen L., Wei W., Wang P. (2020). The role of ubiquitination in tumorigenesis and targeted drug discovery. Signal Transduct. Target. Ther..

[149] Bhattacharjee P., Mazumdar M., Guha D., Sa G., Dhalla N.S., Chakraborti S. (2014). Ubiquitin–Proteasome System in the Hallmarks of Cancer. Role of Proteases in Cellular Dysfunction.

[150] Kitahara O., Furukawa Y., Tanaka T., Kihara C., Ono K., Yanagawa R., E Nita M., Takagi T., Nakamura Y., Tsunoda T. (2001). Alterations of gene expression during colorectal carcinogenesis revealed by cDNA microarrays after laser-capture microdissection of tumor tissues and normal epithelia. Cancer Res..

[151] Lin Y.-M., Furukawa Y., Tsunoda T., Yue C.-T., Yang K.-C., Nakamura Y. (2002). Molecular diagnosis of colorectal tumors by expression profiles of 50 genes expressed differentially in adenomas and carcinomas. Oncogene.

[152] Luo M.J., Lai M.D. (2001). Identification of differentially expressed genes in normal mucosa, adenoma and adenocarcinoma of colon by SSH. World J. Gastroenterol..

[153] Chester K.A., Robson L., Begent R.H., Talbot I.C., Pringle J.H., Primrose L., Macpherson A.J., Boxer G., Southall P., Malcolm A.D. (1989). Identification of a human ribosomal protein mRNA with increased expression in colorectal tumours. Biochim. Biophys. Acta (BBA)-Gene Struct. Expr..

[154] Wang Y., Cheong D., Chan S., Hooi S.C. (2000). Ribosomal protein L7a gene is up-regulated but not fused to the tyrosine kinase receptor as chimeric trk oncogene in human colorectal carcinoma. Int. J. Oncol..

[155] Iizumi Y., Oishi M., Taniguchi T., Goi W., Sowa Y., Sakai T. (2013). The flavonoid apigenin downregulates CDK1 by directly targeting ribosomal protein S9. PLoS ONE.

[156] Huang C.J., Yang S.H., Lee C.L., Cheng Y.C., Tai S.Y., Chien C.C. (2013). Ribosomal protein S27-like in colorectal cancer: A candidate for predicting prognoses. PLoS ONE.

[157] Nieminen T.T., O’donohue M.-F., Wu Y., Lohi H., Scherer S.W., Paterson A.D., Ellonen P., Abdel-Rahman W.M., Valo S., Mecklin J.-P. (2014). Germline Mutation of RPS20, Encoding a Ribosomal Protein, Causes Predisposition to Hereditary Nonpolyposis Colorectal Carcinoma Without DNA Mismatch Repair Deficiency. Gastroenterology.

[158] Broderick P., Dobbins S.E., Chubb D., Kinnersley B., Dunlop M.G., Tomlinson I., Houlston R.S. (2017). Validation of Recently Proposed Colorectal Cancer Susceptibility Gene Variants in an Analysis of Families and Patients—A Systematic Review. Gastroenterology.

[159] González-González M., Sayagués J.M., Muñoz-Bellvís L., Pedreira C.E., de Campos M.L.R., García J., Alcázar J.A., Braz P.F., Galves B.L., González L.M. (2021). Tracking the Antibody Immunome in Sporadic Colorectal Cancer by Using Antigen Self-Assembled Protein Arrays. Cancers.

[160] Kim T.-H., Leslie P., Zhang Y. (2014). Ribosomal proteins as unrevealed caretakers for cellular stress and genomic instability. Oncotarget.

[161] Coppolino M.G., Woodside M.J., Demaurex N., Grinstein S., St-Arnaud R., Dedhar S. (1997). Calreticulin is essential for integrin-mediated calcium signalling and cell adhesion. Nature.

[162] Michalak M., Groenendyk J., Szabo E., Gold L.I., Opas M. (2009). Calreticulin, a multi-process calcium-buffering chaperone of the endoplasmic reticulum. Biochem. J..

[163] Nakamura K., Zuppini A., Arnaudeau S., Lynch J., Ahsan I., Krause R., Papp S., De Smedt H., Parys J.B., Müller-Esterl W. (2001). Functional specialization of calreticulin domains. J. Cell Biol..

[164] Li Y., Liu X., Chen H., Xie P., Ma R., He J., Zhang H. (2021). Bioinformatics analysis for the role of CALR in human cancers. PLoS ONE.

[165] Papp S., Fadel M.P., Kim H., McCulloch C.A., Opas M. (2007). Calreticulin Affects Fibronectin-based Cell-Substratum Adhesion via the Regulation of c-Src Activity. J. Biol. Chem..

[166] Alfonso P., Nunez A., Madoz-Gurpide J., Lombardia L., Sanchez L., Casal J.I. (2005). Proteomic expression analysis of colorectal cancer by two-dimensional differential gel electrophoresis. Proteomics.

[167] Vougas K., Gaitanarou E., Marinos E., Kittas C., Voloudakis-Baltatzis I.E. (2008). Two-dimensional electrophoresis and immunohistochemical study of calreticulin in colorectal adenocarcinoma and mirror biopsies. Off. J. Balk. Union Oncol..

[168] Kaida A., Yamamoto S., Parrales A., Young E.D., Ranjan A., Alalem M.A., Morita K.-I., Oikawa Y., Harada H., Ikeda T. (2021). DNAJA1 promotes cancer metastasis through interaction with mutant p53. Oncogene.

[169] Mattoo R.U.H., Sharma S.K., Priya S., Finka A., Goloubinoff P. (2013). Hsp110 Is a Bona Fide Chaperone Using ATP to Unfold Stable Misfolded Polypeptides and Reciprocally Collaborate with Hsp70 to Solubilize Protein Aggregates. J. Biol. Chem..

[170] Javid H., Hashemian P., Yazdani S., Sharbaf Mashhad A., Karimi-Shahri M. (2022). The role of heat shock proteins in metastatic colorectal cancer: A review. J. Cell. Biochem..

[171] Haase M., Fitze G. (2016). HSP90AB1: Helping the good and the bad. Gene.

[172] Whitesell L., Lindquist S.L. (2005). HSP90 and the chaperoning of cancer. Nat. Rev. Cancer.

[173] Peng C., Zhao F., Li H., Li L., Yang Y., Liu F. (2022). HSP90 mediates the connection of multiple programmed cell death in diseases. Cell Death Dis..

[174] Streicher J.M. (2019). The Role of Heat Shock Proteins in Regulating Receptor Signal Transduction. Mol. Pharmacol..

[175] Tutar Y., Naureen H., Farooqi A.A., Farooqi A.A., Qureshi M.Z., Sabitaliyevich U.Y. (2022). Chapter 13—Heat shock proteins in tumor progression and metastasis. Unraveling the Complexities of Metastasis.

[176] Yu N., Kakunda M., Pham V., Lill J.R., Du P., Wongchenko M., Yan Y., Firestein R., Huang X. (2015). HSP105 Recruits Protein Phosphatase 2A To Dephosphorylate β-Catenin. Mol. Cell. Biol..

[177] Berthenet K., Bokhari A., Lagrange A., Marcion G., Boudesco C., Causse S., De Thonel A., Svrcek M., Goloudina A.R., Dumont S. (2017). HSP110 promotes colorectal cancer growth through STAT3 activation. Oncogene.

[178] Wang W., Wei J., Zhang H., Zheng X., Zhou H., Luo Y., Yang J., Deng Q., Huang S., Fu Z. (2021). PRDX2 promotes the proliferation of colorectal cancer cells by increasing the ubiquitinated degradation of p53. Cell Death Dis..

[179] Lu W., Fu Z., Wang H., Feng J., Wei J., Guo J. (2014). Peroxiredoxin 2 knockdown by RNA interference inhibits the growth of colorectal cancer cells by downregulating Wnt/beta-catenin signaling. Cancer Lett..

[180] Lu W., Fu Z., Wang H., Feng J., Wei J., Guo J. (2014). Peroxiredoxin 2 is upregulated in colorectal cancer and contributes to colorectal cancer cells’ survival by protecting cells from oxidative stress. Mol. Cell. Biochem..

[181] Feng J., Fu Z., Guo J., Lu W., Wen K., Chen W., Wang H., Wei J., Zhang S. (2014). Overexpression of peroxiredoxin 2 inhibits TGF-β1-induced epithelial-mesenchymal transition and cell migration in colorectal cancer. Mol. Med. Rep..

[182] Zhao J., Lin X., Meng D., Zeng L., Zhuang R., Huang S., Lv W., Hu J. (2020). Nrf2 Mediates Metabolic Reprogramming in Non-Small Cell Lung Cancer. Front. Oncol..

[183] Mitsuishi Y., Taguchi K., Kawatani Y., Shibata T., Nukiwa T., Aburatani H., Yamamoto M., Motohashi H. (2012). Nrf2 Redirects Glucose and Glutamine into Anabolic Pathways in Metabolic Reprogramming. Cancer Cell.

[184] Qiu Y., Cai G., Zhou B., Li D., Zhao A., Xie G., Li H., Cai S., Xie D., Huang C. (2014). A distinct metabolic signature of human colorectal cancer with prognostic potential. Clin. Cancer Res..

[185] Li M., Zhao X., Yong H., Xu J., Qu P., Qiao S., Hou P., Li Z., Chu S., Zheng J. (2022). Transketolase promotes colorectal cancer metastasis through regulating AKT phosphorylation. Cell Death Dis..

[186] Zhang D.D. (2006). Mechanistic Studies of the Nrf2-Keap1 Signaling Pathway. Drug Metab. Rev..

[187] Taguchi K., Motohashi H., Yamamoto M. (2011). Molecular mechanisms of the Keap1–Nrf2 pathway in stress response and cancer evolution. Genes Cells.

[188] Baird L., Yamamoto M. (2020). The Molecular Mechanisms Regulating the KEAP1-NRF2 Pathway. Mol. Cell. Biol..

[189] Song M.-Y., Lee D.-Y., Chun K.-S., Kim E.-H. (2021). The Role of NRF2/KEAP1 Signaling Pathway in Cancer Metabolism. Int. J. Mol. Sci..

[190] Wang B., Liu K., Lin H.Y., Bellam N., Ling S., Lin W.C. (2010). 14-3-3Tau regulates ubiquitin-independent proteasomal degradation of p21, a novel mechanism of p21 downregulation in breast cancer. Mol. Cell Biol..

[191] Kästle M., Grune T., Grune T. (2012). Chapter 4—Interactions of the Proteasomal System with Chaperones: Protein Triage and Protein Quality control. Progress in Molecular Biology and Translational Science.

[192] Berthold J., Schenková K., Ramos S., Miura Y., Furukawa M., Aspenström P., Rivero F. (2008). Characterization of RhoBTB-dependent Cul3 ubiquitin ligase complexes—Evidence for an autoregulatory mechanism. Exp. Cell Res..

[193] Wang X., Dong L., Cheng J., Verdine G.L., Lin A., Chu Q. (2022). Targeted β-catenin ubiquitination and degradation by multifunctional stapled peptides. J. Pept. Sci..

[194] Patel J., Tripathi E., Sobti R.C., Lal S.K., Goyal R.K. (2023). Targeting the Ubiquitin Machinery for Cancer Therapeutics. Drug Repurposing for Emerging Infectious Diseases and Cancer.

[195] Ma L., Li X., Zhao X., Sun H., Kong F., Li Y., Sui Y., Xu F. (2021). Oxaliplatin promotes siMAD2L2induced apoptosis in colon cancer cells. Mol. Med. Rep..

[196] Koohini Z., Koohini Z., Teimourian S. (2019). Slit/Robo Signaling Pathway in Cancer, a New Stand Point for Cancer Treatment. Pathol. Oncol. Res..

[197] Jiang Z., Liang G., Xiao Y., Qin T., Chen X., Wu E., Ma Q., Wang Z. (2019). Targeting the SLIT/ROBO pathway in tumor progression: Molecular mechanisms and therapeutic perspectives. Ther. Adv. Med. Oncol..

[198] Kong R., Yi F., Wen P., Liu J., Chen X., Ren J., Li X., Shang Y., Nie Y., Wu K. (2015). Myo9b is a key player in SLIT/ROBO-mediated lung tumor suppression. J. Clin. Investig..

[199] Wang B., Xiao Y., Ding B.-B., Zhang N., Yuan X.-B., Gui L., Qian K.-X., Duan S., Chen Z., Rao Y. (2003). Induction of tumor angiogenesis by Slit-Robo signaling and inhibition of cancer growth by blocking Robo activity. Cancer Cell.

[200] Zhou W.J., Geng Z.H., Chi S., Zhang W., Niu X.F., Lan S.J., Ma L., Yang X., Wang L.J., Ding Y.Q. (2011). Slit-Robo signaling induces malignant transformation through Hakai-mediated E-cadherin degradation during colorectal epithelial cell carcinogenesis. Cell Res..

[201] Dickinson R.E., Fegan K.S., Ren X., Hillier S.G., Duncan W.C. (2011). Glucocorticoid Regulation of SLIT/ROBO Tumour Suppressor Genes in the Ovarian Surface Epithelium and Ovarian Cancer Cells. PLoS ONE.

[202] Prasad A., Fernandis A.Z., Rao Y., Ganju R.K. (2004). Slit Protein-mediated Inhibition of CXCR4-induced Chemotactic and Chemoinvasive Signaling Pathways in Breast Cancer Cells. J. Biol. Chem..

[203] Wang Z., Hou Y., Guo X., van der Voet M., Boxem M., Dixon J.E., Chisholm A.D., Jin Y. (2013). The EBAX-type Cullin-RING E3 Ligase and Hsp90 Guard the Protein Quality of the SAX-3/Robo Receptor in Developing Neurons. Neuron.

[204] Blanco A., Blanco G., Blanco A., Blanco G. (2017). Amino Acid Metabolism. Medical Biochemistry.

[205] Pavlova N.N., Thompson C.B. (2016). The Emerging Hallmarks of Cancer Metabolism. Cell Metab..

[206] Blanco A., Blanco G., Blanco A., Blanco G. (2017). Integration and Regulation of Metabolism. Medical Biochemistry.

[207] Kerk S.A., Lin L., Myers A.L., Sutton D.J., Andren A., Sajjakulnukit P., Zhang L., Zhang Y., Jiménez J.A., Nelson B.S. (2022). Metabolic requirement for GOT2 in pancreatic cancer depends on environmental context. eLife.

[208] Birsoy K., Wang T., Chen W.W., Freinkman E., Abu-Remaileh M., Sabatini D.M. (2015). An Essential Role of the Mitochondrial Electron Transport Chain in Cell Proliferation Is to Enable Aspartate Synthesis. Cell.

[209] Kerk S.A., Garcia-Bermudez J., Birsoy K., Sherman M.H., Shah Y.M., Lyssiotis C.A. (2023). Spotlight on GOT2 in Cancer Metabolism. Onco. Targets Ther..

[210] Du F., Chen J., Liu H., Cai Y., Cao T., Han W., Yi X., Qian M., Tian D., Nie Y. (2019). SOX12 promotes colorectal cancer cell proliferation and metastasis by regulating asparagine synthesis. Cell Death Dis..

[211] Li Z., Li Y., Tang M., Peng B., Lu X., Yang Q., Zhu Q., Hou T., Li M., Liu C. (2018). Destabilization of linker histone H1.2 is essential for ATM activation and DNA damage repair. Cell Res..

[212] Okamura H., Yoshida K., Amorim B.R., Haneji T. (2008). Histone H1.2 is translocated to mitochondria and associates with bak in bleomycin-induced apoptotic cells. J. Cell. Biochem..

[213] Wang Q., Chen Y., Xie Y., Yang D., Sun Y., Yuan Y., Chen H., Zhang Y., Huang K., Zheng L. (2022). Histone H1.2 promotes hepatocarcinogenesis by regulating signal transducer and activator of transcription 3 signaling. Cancer Sci..

[214] Konishi A., Shimizu S., Hirota J., Takao T., Fan Y., Matsuoka Y., Zhang L., Yoneda Y., Fujii Y., Skoultchi A.I. (2003). Involvement of Histone H1.2 in Apoptosis Induced by DNA Double-Strand Breaks. Cell.

[215] Schnetler R., Fanucchi S., Moldoveanu T., Koorsen G. (2020). Linker Histone H1.2 Directly Activates BAK through the K/RVVKP Motif on the C-Terminal Domain. Biochemistry.

[216] Lai S., Jia J., Cao X., Zhou P.-K., Gao S. (2022). Molecular and Cellular Functions of the Linker Histone H1.2. Front. Cell Dev. Biol..

[217] Medrzycki M., Zhang Y., Zhang W., Cao K., Pan C., Lailler N., McDonald J.F., Bouhassira E.E., Fan Y. (2014). Histone H1.3 Suppresses H19 Noncoding RNA Expression and Cell Growth of Ovarian Cancer Cells. Cancer Res..

[218] Armeev G.A., Kniazeva A.S., Komarova G.A., Kirpichnikov M.P., Shaytan A.K. (2021). Histone dynamics mediate DNA unwrapping and sliding in nucleosomes. Nat. Commun..

[219] Hsu C.-L., Chong S.Y., Lin C.-Y., Kao C.-F. (2021). Histone dynamics during DNA replication stress. J. Biomed. Sci..

[220] Prendergast L., Reinberg D. (2021). The missing linker: Emerging trends for H1 variant-specific functions. Genes Dev..

[221] Healton S.E., Pinto H.D., Mishra L.N., Hamilton G.A., Wheat J.C., Swist-Rosowska K., Shukeir N., Dou Y., Steidl U., Jenuwein T. (2020). H1 linker histones silence repetitive elements by promoting both histone H3K9 methylation and chromatin compaction. Proc. Natl. Acad. Sci. USA.

[222] Viéitez C., Martínez-Cebrián G., Solé C., Böttcher R., Potel C.M., Savitski M.M., Onnebo S., Fabregat M., Shilatifard A., Posas F. (2020). A genetic analysis reveals novel histone residues required for transcriptional reprogramming upon stress. Nucleic Acids Res..

[223] Aricthota S., Rana P.P., Haldar D. (2022). Histone acetylation dynamics in repair of DNA double-strand breaks. Front. Genet..

[224] Roberts A.J., Kon T., Knight P.J., Sutoh K., Burgess S.A. (2013). Functions and mechanics of dynein motor proteins. Nat. Rev. Mol. Cell Biol..

[225] Xiang X., Qiu R., Yao X., Arst H.N., Peñalva M.A., Zhang J. (2015). Cytoplasmic dynein and early endosome transport. Cell Mol. Life Sci..

[226] Garrett C.A., Barri M., Kuta A., Soura V., Deng W., Fisher E.M., Schiavo G., Hafezparast M. (2014). DYNC1H1 mutation alters transport kinetics and ERK1/2-cFos signalling in a mouse model of distal spinal muscular atrophy. Brain.

[227] Jeger J.L. (2020). Endosomes, lysosomes, and the role of endosomal and lysosomal biogenesis in cancer development. Mol. Biol. Rep..

[228] Granger E., McNee G., Allan V., Woodman P. (2014). The role of the cytoskeleton and molecular motors in endosomal dynamics. Semin. Cell Dev. Biol..

[229] Scott C.C., Vacca F., Gruenberg J. (2014). Endosome maturation, transport and functions. Semin. Cell Dev. Biol..

[230] Ahlstedt B.A., Ganji R., Raman M. (2022). The functional importance of VCP to maintaining cellular protein homeostasis. Biochem. Soc. Trans..

[231] van den Boom J., Meyer H. (2018). VCP/p97-Mediated Unfolding as a Principle in Protein Homeostasis and Signaling. Mol. Cell.

[232] Song C., Wang Q., Song C., Rogers T.J. (2015). Valosin-containing protein (VCP/p97) is capable of unfolding polyubiquitinated proteins through its ATPase domains. Biochem. Biophys. Res. Commun..

[233] Hemion C., Flammer J., Neutzner A. (2014). Quality control of oxidatively damaged mitochondrial proteins is mediated by p97 and the proteasome. Free. Radic. Biol. Med..

[234] Antoine-Bertrand J., Ghogha A., Luangrath V., Bedford F.K., Lamarche-Vane N. (2011). The activation of ezrin-radixin-moesin proteins is regulated by netrin-1 through Src kinase and RhoA/Rho kinase activities and mediates netrin-1-induced axon outgrowth. Mol. Biol. Cell..

[235] Çelik H., Bulut G., Han J., Graham G.T., Minas T.Z., Conn E.J., Hong S.-H., Pauly G.T., Hayran M., Li X. (2016). Ezrin Inhibition Up-regulates Stress Response Gene Expression. J. Biol. Chem..

[236] Mintz C.D., Carcea I., McNickle D.G., Dickson T.C., Ge Y., Salton S.R., Benson D.L. (2008). ERM proteins regulate growth cone responses to, Sema3A. J. Comp. Neurol..

[237] Bagci H., Sriskandarajah N., Robert A., Boulais J., Elkholi I.E., Tran V., Lin Z.Y., Thibault M.P., Dubé N., Faubert D. (2020). Mapping the proximity interaction network of the Rho-family GTPases reveals signalling pathways and regulatory mechanisms. Nat. Cell Biol..

[238] Gallo G. (2008). Semaphorin 3A inhibits ERM protein phosphorylation in growth cone filopodia through inactivation of PI3K. Dev. Neurobiol..

[239] Riento K., Ridley A.J. (2003). ROCKs: Multifunctional kinases in cell behaviour. Nat. Rev. Mol. Cell Biol..

[240] Lee P.J., Yang S., Sun Y., Guo J.U. (2021). Regulation of nonsense-mediated mRNA decay in neural development and disease. J. Mol. Cell Biol..

[241] Villa N., Fraser C.S., Parsyan A. (2014). Mechanism of Translation in Eukaryotes. Translation and Its Regulation in Cancer Biology and Medicine.

[242] Negrutskii B.S., Shalak V.F., Novosylna O.V., Porubleva L.V., Lozhko D.M., El’skaya A.V. (2023). The eEF1 family of mammalian translation elongation factors. BBA Adv..

[243] Hassan M.K., Kumar D., Naik M., Dixit M. (2018). The expression profile and prognostic significance of eukaryotic translation elongation factors in different cancers. PLoS ONE.

[244] Chi K., Jones D.V., Frazier M.L. (1992). Expression of an elongation factor 1 gamma-related sequence in adenocarcinomas of the colon. Gastroenterology.

[245] Colak D., Ji S.J., Porse B.T., Jaffrey S.R. (2013). Regulation of axon guidance by compartmentalized nonsense-mediated mRNA decay. Cell.

[246] Kadlec J., Izaurralde E., Cusack S. (2004). The structural basis for the interaction between nonsense-mediated mRNA decay factors UPF2 and UPF3. Nat. Struct. Mol. Biol..

[247] Friocourt F., Chédotal A. (2017). The Robo3 receptor, a key player in the development, evolution, and function of commissural systems. Dev. Neurobiol..

[248] Farabaugh P.J., Schaechter M. (2009). Translational Control and Fidelity. Encyclopedia of Microbiology.

[249] Segev N., Gerst J.E. (2018). Specialized Ribosomes and Specific Ribosomal Protein Paralogs Control Translation of Mitochondrial Proteins. J. Cell Biol..

[250] Zhou X., Liao W.J., Liao J.M., Liao P., Lu H. (2015). Ribosomal proteins: Functions beyond the ribosome. J. Mol. Cell Biol..

[251] Kumari A., Kumari A. (2023). Chapter 24—Translation. Sweet Biochemistry.

[252] Puria R., Rohilla S., Kaur S., Kumar V. (2021). Chapter 9—Translation—Process and control. Emerging Concepts in Ribosome Structure, Biogenesis, and Function.

[253] Hu X., Li J., Fu M., Zhao X., Wang W. (2021). The JAK/STAT signaling pathway: From bench to clinic. Signal Transduct. Target. Ther..

[254] Bharadwaj U., Kasembeli M.M., Robinson P., Tweardy D.J. (2020). Targeting Janus Kinases and Signal Transducer and Activator of Transcription 3 to Treat Inflammation, Fibrosis, and Cancer: Rationale, Progress, and Caution. Pharmacol. Rev..

[255] Abd El-Fattah E.E., Zakaria A.Y. (2022). Targeting HSP47 and HSP70: Promising therapeutic approaches in liver fibrosis management. J. Transl. Med..

[256] Morris R., Kershaw N.J., Babon J.J. (2018). The molecular details of cytokine signaling via the JAK/STAT pathway. Protein Sci..

[257] Shan Y., Cortopassi G. (2016). Mitochondrial Hspa9/Mortalin regulates erythroid differentiation via iron-sulfur cluster assembly. Mitochondrion.

[258] Alfadhel M., Nashabat M., Ali Q.A., Hundallah K. (2017). Mitochondrial iron-sulfur cluster biogenesis from molecular understanding to clinical disease. Neurosci. J..

[259] Seif F., Khoshmirsafa M., Aazami H., Mohsenzadegan M., Sedighi G., Bahar M. (2017). The role of JAK-STAT signaling pathway and its regulators in the fate of T helper cells. Cell Commun. Signal..

[260] Ross S.H., Rollings C., Anderson K.E., Hawkins P.T., Stephens L.R., Cantrell D.A. (2016). Phosphoproteomic Analyses of Interleukin 2 Signaling Reveal Integrated JAK Kinase-Dependent and -Independent Networks in CD8+ T Cells. Immunity.

[261] Zareifard A., Beaudry F., Ndiaye K. (2023). Janus Kinase 3 phosphorylation and the JAK/STAT pathway are positively modulated by follicle-stimulating hormone (FSH) in bovine granulosa cells. BMC Mol. Cell Biol..

[262] Koide N., Kasamatsu A., Endo-Sakamoto Y., Ishida S., Shimizu T., Kimura Y., Miyamoto I., Yoshimura S., Shiiba M., Tanzawa H. (2017). Evidence for Critical Role of Lymphocyte Cytosolic Protein 1 in Oral Cancer. Sci. Rep..

[263] Thomas S.J., Snowden J.A., Zeidler M.P., Danson S.J. (2015). The role of JAK/STAT signalling in the pathogenesis, prognosis and treatment of solid tumours. Br. J. Cancer.

[264] Cen K., Lu C. (2023). Prognostic and Immune Infiltration Analysis of Transaldolase 1 (TALDO1) in Hepatocellular Carcinoma. Int. J. Gen. Med..

[265] Alfarsi L.H., El Ansari R., Craze M.L., Mohammed O.J., Masisi B.K., Ellis I.O., Rakha E.A., Green A.R. (2021). SLC1A5 co-expression with TALDO1 associates with endocrine therapy failure in estrogen receptor-positive breast cancer. Breast Cancer Res. Treat..

[266] Moriyama T., Tanaka S., Nakayama Y., Fukumoto M., Tsujimura K., Yamada K., Bamba T., Yoneda Y., Fukusaki E., Oka M. (2016). Two isoforms of TALDO1 generated by alternative translational initiation show differential nucleocytoplasmic distribution to regulate the global metabolic network. Sci. Rep..

[267] Popiołkiewicz J., Polkowski K., Skierski J.S., Mazurek A.P. (2005). In vitro toxicity evaluation in the development of new anticancer drugs—Genistein glycosides. Cancer Lett..

[268] Nordin N., Fadaeinasab M., Mohan S., Hashim N.M., Othman R., Karimian H., Iman V., Ramli N., Ali H.M., Majid N.A. (2016). Pulchrin A, a new natural coumarin derivative of Enicosanthellum pulchrum, induces apoptosis in ovarian cancer cells via intrinsic pathway. PLoS ONE.

