# Supplementary material for: Anti-Cancer Mechanisms of Diarylpentanoid MS17 (1,5-Bis(2-hydroxyphenyl)-1,4-pentadiene-3-one) in Human Colon Cancer Cells: A Proteomics Approach
Source: Int J Mol Sci. 2024 Mar 20;25(6):3503. doi: 10.3390/ijms25063503 (PMC10970808; doi:10.3390/ijms25063503)
Supplement: Supplementary file 1 [file ijms-25-03503-s001.zip › ijms-2888041-supplementary.pdf]

Supplementary Table S1. EC_{50} values of MS17 and curcumin in colon cancer cell lines (SW480 and SW620) and lung fibroblast WI38 cells. Curcumin was used as a positive control. The results were shown as mean ± SEM from three independent experiments.

Cell Line	EC_{50} values of compound (μM)		Selective Index	
	MS17	Curcumin	MS17	Curcumin
SW480	4.1 ± 0.38	17.5 ± 2.36	119.5	147.4
SW620	2.5 ± 0.10	13.1 ± 0.23	196	196.9
WI38	4.9 ± 0.29	25.8 ± 0.92		

Supplementary Table S2. 24 upregulated DEPs in MS17-treated SW480 cells upon 8.2 μM treatment for 24 hours.

Protein Class/ Protein Name	Accession Number	Protein Symbol/ Gene Symbol	Significance (-10LogP)	Coverage (%)	#Peptides	#Unique	Group Profile (Ratio)	Avg. Mass
Calcium signaling								
Protein S100-A6	P06703	S10A6/S100A6	13.24	64	6	6	1.00:1.32	10180
Cytoskeletal protein								
Actin cytoplasmic 1	P60709*	ACTB/ ACTB	13.2	5	1	1	1.00:1.32	41737
Chaperones								
Endoplasmic reticulum chaperone BiP	P11021	BIP/HSPA5	20.3	29	17	17	1.00:1.54	72333
10 kDa heat shock protein mitochondrial	P61604	CH10/HSPE1	47.7	55	6	6	1.00:2.68	10932
Heat shock 70 kDa protein 1A	P0DMV8*	HS71A/HSPA1A	77.24	16	8	8	1.00:9.15	70052
Heat shock 70 kDa protein 1B	P0DMV9*	HS71B/HSPA1B	77.24	16	8	8	1.00:9.15	70052
Heat shock protein HSP 90-alpha	P07900	HS90A/ HSP90AA1	106.5	19	14	14	1.00:2.48	84660
Heat shock protein HSP 90-alpha A2	Q14568	HS902/ HSP90AA2P	106.5	10	3	3	1.00:2.17	39365
Heat shock protein HSP 90-alpha A4	Q58FG1	HS904/ HSP90AA4P	106.5	5	2	2	1.00:1.81	47712
Serpin H1	P50454	SERPINH1/ SERPINH1	18.71	13	4	4	1.00:2.40	46441
Chromatin-regulatory								
Putative high mobility group protein B1-like 1	B2RPK0	HGB1A/ HMGB1P1	15.28	7	2	2	1.00:1.98	24238
High mobility group protein B1	P09429	HMGB1/ HMGB1	15.28	7	2	2	1.00:1.98	24894
Metabolic enzyme								
Pyruvate kinase PKM	P14618	KPYM/PKM	30.9	44	20	20	1.00:1.44	57937
Pyruvate kinase PKLR	P30613	KPYR/PKLR	30.9	2	1	1	1.00:1.36	61830
Nuclear membrane protein								
Neuron navigator 3	Q8IVL0	NAV3/NAV3	47.7	0	1	1	1.00:2.07	255646

Ribosomal protein								
60S ribosomal protein L7a	P62424*	RL7A/ RPL7A	36.95	19	5	5	1.00:1.48	29996
Ubiquitin-40S ribosomal protein S27a	P62979	RS27A/RPS27A	35.92	28	4	4	1.00:2.63	17965
Ubiquitin-60S ribosomal protein L40	P62987	RL40/UBA52	35.92	39	5	5	1.00:2.63	14728
Transport protein								
ATP-binding cassette sub-family A member 13	Q86UQ4	ABCAD/ABCA13	106.5	0	1	1	1.00:2.00	576166
GTP-binding nuclear protein Ran	P62826	RAN/RAN	14.76	25	5	5	1.00:1.34	24423
Ubiquitin-protein ligase								
Polyubiquitin-B	P0CG47	UBB/UBB	35.92	19	4	4	1.00:2.63	25762
Polyubiquitin-C	P0CG48	UBC/UBC	35.92	6	4	4	1.00:2.63	77039

*DEPs with * overlapped with SW620 cells.*

Supplementary Table S3. 92 DEPs in MS17-treated SW620 cells upon 5μM treatment for 24 hours.

Protein Class/ Protein Name	Accession Number	Protein Symbol/ Gene Symbol	Significance (-10LogP)	Coverage (%)	#Peptides	#Unique	Group Profile (Ratio)	Avg. Mass
Calcium signaling								
Calmodulin-1	P0DP23	CALM1/CALM1	20.25	36	3	3	1.00:6.06	16838
Calmodulin-2	P0DP24	CALM2/CALM2	20.25	36	3	3	1.00:6.06	16838
Calmodulin-3	P0DP25	CALM3/CALM3	20.25	36	3	3	1.00:6.06	16838
Annexin A1	P04083	ANXA1/ANXA1	51.62	24	7	7	1.00:6.38	38714
Calmodulin-like protein 3	P27482	CALL3/CALML3	20.25	5	1	1	1.00:64.00	16891
Chaperone								
Prefoldin subunit 6	O15212	PFD6/PFDN6	19.01	6	1	1	1.00:64.00	14583
Heat shock 70 kDa protein 1A	P0DMV8*	HS71A/HSPA1A	20.93	4	5	5	1.00:4.05	70052
Heat shock 70 kDa protein 1B	P0DMV9*	HS71B/HSPA1B	20.93	4	5	5	1.00:4.05	70052
Nucleophosmin	P06748	NPM/NPM1	15.09	39	11	11	1.00:1.52	32575
Heat shock protein HSP 90-beta	P08238	HS90B/HSP90AB1	142.39	1	1	1	1.00:5.34	83264
Calreticulin	P27797	CALR/CALR	16.16	4	2	2	1.00:0.73	48142
DnaJ homolog subfamily A member 1	P31689	DNJA1/DNAJA1	38.15	13	3	3	1.00:12.32	44868
Stress-70 protein, mitochondrial	P38646	GRP75/HSPA9	30.79	5	3	3	1.00:0.63	73681
T-complex protein 1 subunit theta	P50990	TCPQ/CCT8	46.29	10	6	6	1.00:1.80	59621
Heat shock protein 75 kDa mitochondrial	Q12931	TRAP1/TRAP1	22.03	4	1	1	1.00:15.33	80110

Putative heat shock protein HSP 90-beta-3	Q58FF7	H90B3/HSP90AB3P	142.39	2	1	1	1.00:5.34	68325
Heat shock protein 105 kDa	Q92598	HS105/HSPH1	49.22	7	5	5	1.00:64.00	96865
Parkinson disease protein 7	Q99497	PARK7/PARK7	35.02	47	8	8	1.00:1.65	19891
T-complex protein 1 subunit eta	Q99832	TCPH/CCT7	15	12	6	6	1.00:2.98	59367
Chromatin-regulatory								
Histone H1.3	P16402	H13/H1-3	13.46	7	1	1	1.00:0.21	22350
Histone H1.2	P16403	H12/H1-2	66.24	12	2	2	1.00:0.31	21365
Cytoskeletal protein								
Keratin type II cytoskeletal 7	P08729	K2C7/KRT7	28.73	4	2	2	1.00:0.09	51386
Alpha-actinin-1	P12814	ACTN1/ACTN1	29.32	4	1	1	1.00:8.84	103058
Plastin-2	P13796	PLSL/LCP1	17.67	6	2	2	1.00:2.42	70289
Stathmin	P16949	STMN1/STMN1	21.54	19	3	3	1.00:0.58	17302
Vinculin	P18206	VINC/VCL	13.08	7	4	4	1.00:3.12	123799
Moesin	P26038	MOES/MSN	15.3	10	6	6	1.00:3.19	67820
Actin cytoplasmic 1	P60709*	ACTB/ ACTB	56.91	5	1	1	1.00:0.46	41737
Thymosin beta-4	P62328	TYB4/TMSB4X	200	16	1	1	1.00:0.31	5053
Thymosin beta-10	P63313	TYB10/TMSB10	200	16	1	1	1.00:0.31	5026
LIM and SH3 domain protein 1	Q14847	LASP1/LASP1	13.46	22	6	6	1.00:0.42	29717
DNA-binding								
Proliferating cell nuclear antigen	P12004	PCNA/PCNA	200	15	5	5	1.00:5.27	28769
Transcription factor BTF3	P20290	BTF3/BTF3	23.93	7	2	2	1.00:0.06	22168
Catenin beta-1	P35222	CTNB1/CTNNB1	31.62	7	3	3	1.00:4.29	85497
Cytoplasmic dynein	Q14204	DYHC1/DYNC1H1	34.98	1	2	2	1.00:4.24	532412
Metabolic enzyme								
Aspartate aminotransferase mitochondrial	P00505	AATM/GOT2	29.2	4	2	2	1.00:2.09	47518
Inosine-5'-monophosphate dehydrogenase 2	P12268	IMDH2/IMPDH2	51.27	2	1	1	1.00:0.54	55805
Phosphoglycerate mutase 1	P18669	PGAM1/PGAM1	77.72	7	1	1	1.00:2.19	28804
Transketolase	P29401	TKT/TKT	29.33	4	2	2	1.00:2.39	67878
Peroxiredoxin-2	P32119	PRDX2/PRDX2	16.56	17	3	3	1.00:1.41	21892
Transaldolase	P37837	TALDO/TALDO1	15.61	6	2	2	1.00:0.30	37540
Electron transfer flavoprotein subunit beta	P38117	ETFB/ETFB	15.38	7	2	2	1.00:4.17	27844

Triosephosphate isomerase	P60174	TPIS/TPI1	26.09	47	9	9	1.00:0.61	26669
Dihydropyrimidinase-related protein 2	Q16555	DPYL2/DPYSL2	13.03	1	1	1	1.00:0.49	62294
Probable phosphoglycerate mutase 4	Q8N0Y7	PGAM4/PGAM4	77.72	7	1	1	1.00:2.19	28777
Nuclear protein								
Prothymosin alpha	P06454	PTMA/PTMA	200	13	1	1	1.00:0.28	12203
Apoptosis inhibitor 5	Q9BZZ5	API5/API5	47.73	9	2	2	1.00:1.93	59005
Peripheral membrane protein								
Clathrin heavy chain 2	P53675	CLH2/CLTCL1	60.29	1	1	1	1.00:1.74	187029
Clathrin heavy chain 1	Q00610	CLH1/CLTC	60.29	3	3	3	1.00:2.11	191613
Ubiquitin-protein ligase								
Proteasome subunit alpha type 1	P25786	PSA1/PSMA1	19.81	3	1	1	1.00:64.00	29556
26S proteasome non-ATPase regulatory subunit 13	Q9UNM6	PSD13/PSMD13	19.84	6	2	2	1.00:14.82	42946
Ribosomal protein								
60S acidic ribosomal protein P2	P05387	RLA2/RPLP2	14.95	46	3	3	1.00:0.37	11665
40S ribosomal protein S12	P25398	RS12/RPS12	22.29	24	2	2	1.00:3.53	14515
40S ribosomal protein S27	P42677	RS27/RPS27	200	10	1	1	1.00:4.90	9461
40S ribosomal protein S9	P46781	RS9/RPS9	36.93	28	6	6	1.00:3.66	22591
40S ribosomal protein S10	P46783	RS10/RPS10	34.17	11	2	2	1.00:0.06	18898
40S ribosomal protein S20	P60866	RS20/RPS20	31.23	15	2	2	1.00:9.14	13373
40S ribosomal protein S3a	P61247	RS3A/RPS3A	15.4	3	1	1	1.00:1.35	29945
60S ribosomal protein L26	P61254	RL26/RPL26	13.63	12	2	2	1.00:4.63	17258
40S ribosomal protein S11	P62280	RS11/RPS11	66.24	20	4	4	1.00:2.05	18431
60S ribosomal protein L7a	P62424*	RL7A/ RPL7A	36.44	20	5	5	1.00:0.60	29996
40S ribosomal protein S6	P62753	RS6/RPS6	16.53	12	3	3	1.00:3.74	28681

40S ribosomal protein S15	P62841	RS15/RPS15	32.42	15	1	1	1.00:2.40	17040
40S ribosomal protein S28	P62857	RS28/RPS28	16.26	33	2	2	1.00:0.46	7841
60S ribosomal protein L10a	P62906	RL10A/RPL10A	127.29	4	1	1	1.00:2.77	24831
60S ribosomal protein L8	P62917	RL8/RPL8	14.42	4	1	1	1.00:4.58	28025
60S ribosomal protein L6	Q02878	RL6/RPL6	25.24	14	4	4	1.00:1.51	32728
40S ribosomal protein S27-like	Q71UM5	RS27L/RPS27L	200	10	1	1	1.00:4.90	9477
60S ribosomal protein L26-like 1	Q9UNX3	RL26L/RPL26L1	13.63	12	2	2	1.00:4.63	17256
RNA-binding								
Eukaryotic translation initiation factor 3 subunit F	O00303	EIF3F/EIF3F	150.51	5	1	1	1.00:5.18	37564
Splicing factor proline- and glutamine-rich	P23246	SFPQ/SFPQ	66.28	3	2	2	1.00:3.52	76150
Elongation factor 1-gamma	P26641	EF1G/EEF1G	20.26	19	9	9	1.00:1.58	50119
Heterogeneous nuclear ribonucleoprotein M	P52272	HNRPM/HNRNPM	30.3	5	5	5	1.00:1.58	77516
Heterogeneous nuclear ribonucleoprotein K	P61978	HNRPK/HNRNPK	13.1	26	11	11	1.00:0.59	50976
Eukaryotic translation initiation factor 5A-1	P63241	IF5A1/EIF5A	53.79	41	5	5	1.00:0.53	16832
Serine/arginine-rich splicing factor 3	P84103	SRSF3/SRSF3	33.26	12	2	2	1.00:0.49	19330
Serine/arginine-rich splicing factor 4	Q08170	SRSF4/SRSF4	15.45	3	2	2	1.00:2.85	56678
ATP-dependent RNA helicase A	Q08211	DHX9/DHX9	14.57	4	2	2	1.00:3.20	140958
Serine/arginine-rich splicing factor 5	Q13243	SRSF5/SRSF5	15.45	3	1	1	1.00:1.93	31264
Serine/arginine-rich splicing factor 6	Q13247	SRSF6/SRSF6	15.45	5	2	2	1.00:2.31	39587
Non-POU domain-containing octamer-binding protein	Q15233	NONO/NONO	14.67	10	4	4	1.00:1.51	54232
Poly(rC)-binding protein 1	Q15365	PCBP1/PCBP1	14.11	6	2	2	1.00:4.17	37498
Poly(rC)-binding protein 2	Q15366	PCBP2/PCBP2	19.01	7	2	2	1.00:1.76	38580
Eukaryotic translation initiation factor 5A-1-like	Q6IS14	IF5AL/EIF5AL1	53.79	36	4	4	1.00:0.53	16773
Pre-mRNA-processing-splicing factor 8	Q6P2Q9	PRP8/PRPF8	15.59	1	1	1	1.00:4.93	273599

Plasminogen activator inhibitor 1 RNA-binding protein	Q8NC51	PAIRB/SERBP1	85.16	7	2	2	1.00:0.44	44965
Eukaryotic translation initiation factor 5A-2	Q9GZV4	IF5A2/EIF5A2	53.79	18	3	3	1.00:0.53	16793
Scaffold protein								
Ran-specific GTPase-activating protein	P43487	RANG/RANBP1	128.06	8	2	2	1.00:8.56	23310
Transport protein								
ADP-ribosylation factor 4	P18085	ARF4/ ARF4	27.05	19	3	3	1.00:4.45	20511
Exportin-2	P55060	XPO2/CSE1L	200	11	6	6	1.00:3.54	110417
Transitional endoplasmic reticulum ATPase	P55072	TERA/VCP	17.83	2	1	1	1.00:8.42	89322
Importin subunit beta 1	Q14974	IMB1/KPNB1	17.63	4	3	3	1.00:1.57	97170

*DEPs with * overlapped with SW620 cells.*

Supplementary Table S4. STRING clustering of DEPs and first shell interactors in MS17-treated SW480 cells with the respective clusters and protein classes upon 8.2μM of MS17 treatment for 24 hours.

Protein Cluster/Protein Name	Gene Symbol	Protein Symbol	Protein Class
Cluster 1			
Activator of HSP90 ATPase Activity 1*	AHSA1	AHSA1	Chaperone
HSP90 co-chaperone CDC37*	CDC37	CDC37	Chaperone
Heat shock protein HSP 90-alpha	HSP90AA1	HS90A	Chaperone
Heat shock 70 kDa protein 1A	HSPA1A	HS71A	Chaperone
Heat shock 70 kDa protein 1B	HSPA1B	HS71B	Chaperone
Endoplasmic reticulum chaperone BiP	HSPA5	BIP	Chaperone
10 kDa heat shock protein, mitochondrial	HSPE1	CH10	Chaperone
High mobility group protein B1	HMGB1	HMGB1	Chromatin-regulatory
Actin, cytoplasmic 1	ACTB	ACTB	Cytoskeletal protein
Profilin-1*	PFN1	PFN1	Cytoskeletal protein
Peptidyl-prolyl cis-trans isomerase	FKBP4	FKBP4	Metabolic enzyme
Neuron navigator 3	NAV3	NAV3	Nuclear membrane protein
Nuclear transport factor 2*	NUTF2	NTF2	Transport protein
Serpin H1	SERPINH1	SERPINH1	Chaperone
E3 ubiquitin-protein ligase CHIP*	STUB1	CHIP	Ubiquitin-protein ligase
Cluster 2			
Protein S100-A6	S100A6	S10A6	Calcium signaling
Pyruvate kinase PKLR	PKLR	KPYR	Metabolic enzyme
Pyruvate kinase PKM	PKM	KPYM	Metabolic enzyme
Ribosomal protein L18a*	RPL18A	RL18A	Ribosomal protein
Ribosomal protein L19*	RPL19	RL19	Ribosomal protein
Ribosomal protein L7a	RPL7A	RL7A	Ribosomal protein
Ribosomal protein S12*	RPS12	RS12	Ribosomal protein
Ubiquitin-40S ribosomal protein S27a	RPS27A	RPS27A	Ribosomal protein
Ubiquitin-60S ribosomal protein L40	UBA52	RL40	Ribosomal protein
ATP binding cassette subfamily A member 13	ABCA13	ABCAD	Transport protein

GTP-binding nuclear protein Ran	RAN	RAN	Transport protein
Polyubiquitin-B	UBB	UBB	Ubiquitin-protein ligase
Polyubiquitin-C	UBC	UBC	Ubiquitin-protein ligase
Ubiquitin carboxyl-terminal hydrolase 5*	USP5	USP5	Ubiquitin-protein ligase

Proteins with *: First shell protein interactor determined by STRING Network.

Supplementary Table S5. STRING clustering of 92 DEPs in MS17-treated SW620 cells with the respective clusters and protein classes upon 5μM of MS17 treatment for 24 hours.

Protein Cluster/Protein Name	Gene Symbol	Protein Symbol	Protein Class
Cluster 1			
Transcription factor BTF3*	BTF3	BTF3	DNA-binding
Proliferating cell nuclear antigen	PCNA	PCNA	DNA-binding
60S ribosomal protein L10a	RPL10A	RL10A	Ribosomal protein
60S ribosomal protein L26	RPL26	RL26	Ribosomal protein
Ribosomal protein L26 like 1	RPL26L1	RL26L1	Ribosomal protein
60S ribosomal protein L6	RPL6	RL6	Ribosomal protein
Ribosomal protein L7a	RPL7A	RL7A	Ribosomal protein
60S ribosomal protein L8	RPL8	RL8	Ribosomal protein
60S acidic ribosomal protein P2*	RPLP2	RLA2	Ribosomal protein
40S ribosomal protein S10*	RPS10	RS10	Ribosomal protein
Ribosomal protein S11	RPS11	RS11	Ribosomal protein
Ribosomal protein S12	RPS12	RS12	Ribosomal protein
Ribosomal protein S15	RPS15	RS15	Ribosomal protein
Ribosomal protein S20	RPS20	RS20	Ribosomal protein
40S ribosomal protein S27	RPS27	RS27	Ribosomal protein
Ribosomal protein S27 like	RPS27L	RS27L	Ribosomal protein
Ribosomal protein S28*	RPS28	RS28	Ribosomal protein
40S ribosomal protein S3a	RPS3A	RS3A	Ribosomal protein
40S ribosomal protein S6	RPS6	RS6	Ribosomal protein
Ribosomal protein S9	RPS9	RS9	Ribosomal protein
Plasminogen activator inhibitor 1 RNA-binding protein*	SERBP1	PAIRB	Ribosomal protein
Eukaryotic translation initiation factor 3 subunit F	EIF3F	EIF3F	RNA-binding
Eukaryotic translation initiation factor 5A-1*	EIF5A	IF5A1	RNA-binding
Eukaryotic translation initiation factor 5A-2*	EIF5A2	IF5A2	RNA-binding
Eukaryotic translation initiation factor 5A-1-like*	EIF5AL1	IF5AL	RNA-binding
Electron transfer flavoprotein subunit beta	ETFB	ETFB	Metabolic enzyme
ADP-ribosylation factor 4	ARF4	ARF4	Transport protein
Cluster 2			
Calreticulin*	CALR	CALR	Chaperone
T-complex protein 1 subunit eta	CCT7	TCPH	Chaperone
T-complex protein 1 subunit theta	CCT8	TCPQ	Chaperone
DnaJ homolog subfamily A member 1	DNAJA1	DNAJ1	Chaperone
Heat shock protein HSP 90-beta	HSP90AB1	HS90B	Chaperone

Heat shock 70 kDa protein 1A	HSPA1A	HS71A	Chaperone
Heat shock 70 kDa protein 1B	HSPA1B	HS71B	Chaperone
Stress-70 protein, mitochondrial*	HSPA9	GRP75	Chaperone
Heat shock protein 105 kDa	HSPH1	HS105	Chaperone
Nucleophosmin	NPM1	NPM	Chaperone
Parkinson disease protein 7	PARK7	PARK7	Chaperone
Prefoldin subunit 6	PFDN6	PFD6	Chaperone
Exportin-2	CSE1L	XPO2	Transport protein
Importin subunit beta 1	KPNB1	IMB1	Transport protein
Transitional endoplasmic reticulum ATPase	VCP	TERA	Transport protein
Calmodulin-3	CALM3	CALM3	Calcium signaling
Calmodulin-like protein 3	CALML3	CALL3	Calcium signaling
Proteasome subunit alpha type 1	PSMA1	PSA1	Ubiquitin-protein ligase
26S proteasome non-ATPase regulatory subunit 13	PSMD13	PSD13	Ubiquitin-protein ligase
LIM and SH3 domain protein 1*	LASP1	LASP1	Cytoskeletal protein
Inosine-5'-monophosphate dehydrogenase 2*	IMPDH2	IMDH2	Metabolic enzyme
Elongation factor 1-gamma	EEF1G	EEF1G	RNA-binding
Ran-specific GTPase-activating protein	RANBP1	RANG	Scaffold protein
Cluster 3			
ATP-dependent RNA helicase A	DHX9	DHX9	RNA-binding
Heterogeneous nuclear ribonucleoprotein K*	HNRNPK	HNRPK	RNA-binding
Heterogeneous nuclear ribonucleoprotein M	HNRNPM	HNRPM	RNA-binding
Non-POU domain-containing octamer-binding protein	NONO	NONO	RNA-binding
Poly(rC)-binding protein 1	PCBP1	PCBP1	RNA-binding
Poly(rC)-binding protein 2	PCBP2	PCBP2	RNA-binding
Pre-mRNA-processing-splicing factor 8	PRPF8	PRP8	RNA-binding
Splicing factor, proline- and glutamine-rich	SFPQ	SFPQ	RNA-binding
Serine/arginine-rich splicing factor 3*	SRSF3	SRSF3	RNA-binding
Serine/arginine-rich splicing factor 4	SRSF4	SRSF4	RNA-binding
Serine/arginine-rich splicing factor 5	SRSF5	SRSF5	RNA-binding
Serine/arginine-rich splicing factor 6	SRSF6	SRSF6	RNA-binding
Histone H1.2*	H1.2	H1-2	Chromatin-regulatory
Histone H1.3*	H1.3	H1-3	Chromatin-regulatory
Keratin, type II cytoskeletal 7*	KRT7	K2C7	Cytoskeletal protein
Apoptosis Inhibitor 5	API5	API5	Nuclear protein
Cluster 4			
Actin, cytoplasmic 1*	ACTB	ACTB	Cytoskeletal protein
Alpha-actinin-1	ACTN1	ACTN1	Cytoskeletal protein
Plastin-2	LCP1	PLSL	Cytoskeletal protein
Moesin	MSN	MOES	Cytoskeletal protein
Thymosin beta-10*	TMSB10	TYB10	Cytoskeletal protein
Thymosin beta 4*	TMSB4X	TYB4	Cytoskeletal protein
Vinculin	VCL	VINC	Cytoskeletal protein
Catenin beta 1	CTNNB1	CTNB1	DNA-binding
Cytoplasmic dynein 1 heavy chain 1	DYNC1H1	DYHC1	DNA-binding

Clathrin heavy chain 1	CLTC	CLH1	Peripheral membrane protein
Clathrin heavy chain 2	CLTCL1	CLH2	Peripheral membrane protein
Annexin A1	ANXA1	ANXA1	Calcium signaling
Heat shock protein 75 kDa, mitochondrial	TRAP1	TRAP1	Chaperone
Prothymosin alpha*	PTMA	PTMA	Nuclear protein
Cluster 5			
Dihydropyrimidinase-related protein 2*	DPYSL2	DPYL2	Metabolic enzyme
Aspartate aminotransferase, mitochondrial	GOT2	AATM	Metabolic enzyme
Phosphoglycerate mutase 1	PGAM1	PGAM1	Metabolic enzyme
Phosphoglycerate mutase family member 4	PGAM4	PGAM4	Metabolic enzyme
Peroxiredoxin-2	PRDX2	PRDX2	Metabolic enzyme
Transaldolase*	TALDO1	TALDO	Metabolic enzyme
Transketolase	TKT	TKT	Metabolic enzyme
Triosephosphate isomerase*	TPI1	TPIS	Metabolic enzyme
Calmodulin-1	CALM1	CALM1	Calcium signaling
Calmodulin 2	CALM2	CALM2	Calcium signaling
Stathmin*	STMN1	STMN1	Cytoskeletal protein

*DEPs with *: downregulated in MS17-treated SW620 cells.*
